# Neuronal metabotropic glutamate receptor 8 protects against neurodegeneration in CNS inflammation

**DOI:** 10.1084/jem.20201290

**Published:** 2021-03-04

**Authors:** Marcel S. Woo, Friederike Ufer, Nicola Rothammer, Giovanni Di Liberto, Lars Binkle, Undine Haferkamp, Jana K. Sonner, Jan Broder Engler, Sönke Hornig, Simone Bauer, Ingrid Wagner, Kristof Egervari, Jacob Raber, Robert M. Duvoisin, Ole Pless, Doron Merkler, Manuel A. Friese

**Affiliations:** 1 Institut für Neuroimmunologie und Multiple Sklerose, Zentrum für Molekulare Neurobiologie Hamburg, Universitätsklinikum Hamburg-Eppendorf, Hamburg, Germany; 2 Division of Clinical Pathology, Department of Pathology and Immunology, Geneva Faculty of Medicine, Geneva, Switzerland; 3 Fraunhofer Institute for Translational Medicine and Pharmacology, Hamburg, Germany; 4 Experimentelle Neuropädiatrie, Klinik für Kinder und Jugendmedizin, Universitätsklinikum Hamburg-Eppendorf, Hamburg, Germany; 5 Department of Behavioral Neuroscience, Oregon Health & Science University, Portland, OR; 6 Department of Neurology, Oregon Health & Science University, Portland, OR; 7 Department of Radiation Medicine, Oregon Health & Science University, Portland, OR; 8 Division of Neuroscience, Oregon National Primate Research Center, Oregon Health & Science University, Portland, OR; 9 Department of Chemical Physiology and Biochemistry, Oregon Health & Science University, Portland, OR

## Abstract

Multiple sclerosis (MS) is a chronic inflammatory disease of the central nervous system with continuous neuronal loss. Treatment of clinical progression remains challenging due to lack of insights into inflammation-induced neurodegenerative pathways. Here, we show that an imbalance in the neuronal receptor interactome is driving glutamate excitotoxicity in neurons of MS patients and identify the MS risk–associated metabotropic glutamate receptor 8 (*GRM8*) as a decisive modulator. Mechanistically, GRM8 activation counteracted neuronal cAMP accumulation, thereby directly desensitizing the inositol 1,4,5-trisphosphate receptor (IP3R). This profoundly limited glutamate-induced calcium release from the endoplasmic reticulum and subsequent cell death. Notably, we found *Grm8*-deficient neurons to be more prone to glutamate excitotoxicity, whereas pharmacological activation of GRM8 augmented neuroprotection in mouse and human neurons as well as in a preclinical mouse model of MS. Thus, we demonstrate that GRM8 conveys neuronal resilience to CNS inflammation and is a promising neuroprotective target with broad therapeutic implications.

## Teaser

Systematic analysis of the neuronal receptor interactome in multiple sclerosis brains reveals the metabotropic glutamate receptor 8 as a disbalanced hub. Its activation constitutes a novel treatment strategy to enhance neuronal resilience by inhibiting pathological calcium overload in inflammation-induced neurodegeneration.

## Graphical Abstract

**Figure:**
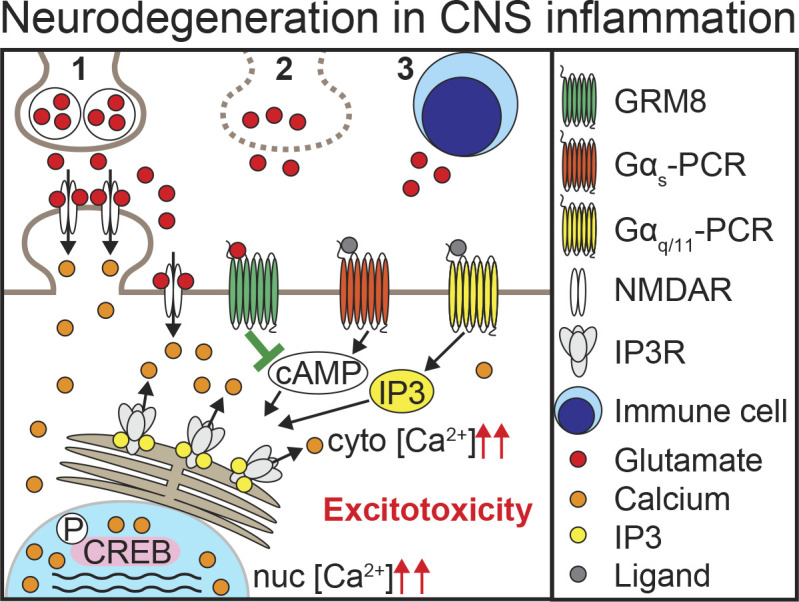

## Introduction

Multiple sclerosis (MS) is the predominant nontraumatic cause of neurological disability in young adults and thereby constitutes a substantial healthcare and socioeconomic burden (Reich et al., 2018). Its pathogenesis has been mostly attributed to an infiltration of autoreactive immune cells into the central nervous system (CNS) with concurrent demyelination and neuroaxonal degeneration (Dendrou et al., 2015). Although immunomodulatory treatments effectively suppress inflammatory relapses of the disease, neurodegeneration is not halted. Therefore, increasing neuronal resilience to inflammatory stress in MS constitutes a major unmet clinical need (Friese et al., 2014).

Neuronal loss in MS and its animal model, experimental autoimmune encephalomyelitis (EAE), is initiated by continuous inflammatory insults. Infiltrating immune cells, together with CNS-resident microglia, releases multiple inflammatory mediators that induce synaptic loss (Di Filippo et al., 2018) and disturb neuroaxonal integrity (Nikić et al., 2011). It has been proposed that production of reactive oxygen and nitrogen species, together with iron deposition, damages neuronal mitochondria with subsequent metabolic failure (Campbell et al., 2011; Stephenson et al., 2014). Disruption of neuronal ion homeostasis (Friese et al., 2007) and aggregation of neuronal proteins might further drive neuroaxonal demise (Schattling et al., 2019). However, identifying druggable targets that specifically induce neuronal resilience has been notoriously difficult due to lack of insights into key modulators of injurious neuronal stress responses or severe adverse effects of their modulation. For example, dysregulated neuronal calcium influx has been proposed to drive neuronal loss in primary and secondary neurodegenerative diseases (Hardingham et al., 2001), but broad inhibition of calcium influx results in significant reduction of neuronal functionality (Yasuda et al., 2017; Rowland et al., 2005). Moreover, only few molecular targets have been identified with neuroprotective properties that are separable from their impact on inflammatory responses, such as the acid-sensing ion channel 1 (Friese et al., 2007), transient receptor potential melastatin 4 (Schattling et al., 2012), the integrated stress response (Stone et al., 2019), nucleocytoplasmic shuttling (Haines et al., 2015), or the mitochondrial matrix protein cyclophilin D (Forte et al., 2007). Therefore, further dissection of neuron-intrinsic mechanisms that are dysregulated in response to inflammation is critical to identify treatment strategies that counteract neurodegeneration.

A pathological feature shared between primary neurodegenerative diseases such as Alzheimer’s disease, Parkinson’s disease, amyotrophic lateral sclerosis (Dong et al., 2009), and MS is neuroinflammation (Ransohoff, 2016) together with elevated glutamate levels in the brain (Srinivasan et al., 2005) and the cerebrospinal fluid (Sarchielli et al., 2003) that likely contributes to neuronal injury. This excessive amount of extracellular glutamate, the main excitatory amino acid, results from intracellular release of dying cells, active secretion by immune cells (Birkner et al., 2020), and impaired glutamate reuptake (Macrez et al., 2016) that collectively induce cell death in neurons by unregulated calcium accumulation. Thus, tight control of glutamate is critical to preserve homeostasis, ensuring neuronal functionality. Central players in this delicate balance are excitatory ionotropic glutamate receptors (iGluRs) and Gα_q/11_-coupled metabotropic glutamate receptors (mGluRs) that are opposed by inhibitory Gα_i_-coupled mGluRs (Reiner and Levitz, 2018). Although blocking iGluRs is protective in EAE (Smith et al., 2000), their clinical use remains challenging due to lack of specificity and severe neuropsychiatric adverse effects (Kalia et al., 2008). Moreover, different approaches to block Gα_q/11_-coupled mGluRs, such as metabotropic glutamate receptor 1 (GRM1) or GRM5, failed to show neuroprotective efficacy in EAE (Sulkowski et al., 2013). While genetic variants of iGluR and mGluR have been associated with MS risk and severity (Baranzini et al., 2009; Briggs et al., 2011), which glutamate receptor signaling network modulates inflammation-induced neurodegeneration remains elusive.

In this study, we set out to investigate neuron-specific stress responses in an inflammatory environment and compared transcriptional signatures and receptor interactome networks of neurons that were exposed to defined stressors with transcriptional responses of neurons in the CNS of MS patients and EAE mice. We demonstrate that glutamate stress signature genes have the strongest enrichment across all MS and EAE datasets and identify the regulatory network of the MS risk–associated inhibitory GRM8 to be robustly enriched in neurons of MS patients. Reasoning that increasing GRM8 activity might be limiting neurodegeneration, we found that pharmacological activation of GRM8 was neuroprotective in mouse neurons in vitro and reduced inflammation-induced neurodegeneration in vivo. Accordingly, *Grm8-*deficient mice showed more severe neurodegeneration during CNS inflammation. Mechanistically, we can show that GRM8 negatively regulates cAMP-dependent sensitization of inositol 1,4,5-trisphosphate (IP3) receptors (IP3Rs), thereby limiting glutamate-induced calcium release from the ER. Importantly, we were able to successfully translate these findings to human MS brains and human induced pluripotent stem cell (hiPSC)–derived neurons. These results support the activation of GRM8 as a broad therapeutic strategy to enhance neuronal resilience by counteracting glutamate excitotoxicity in neurodegeneration.

## Results

### Neuronal glutamate receptor signaling during CNS inflammation

**Figure 1.**
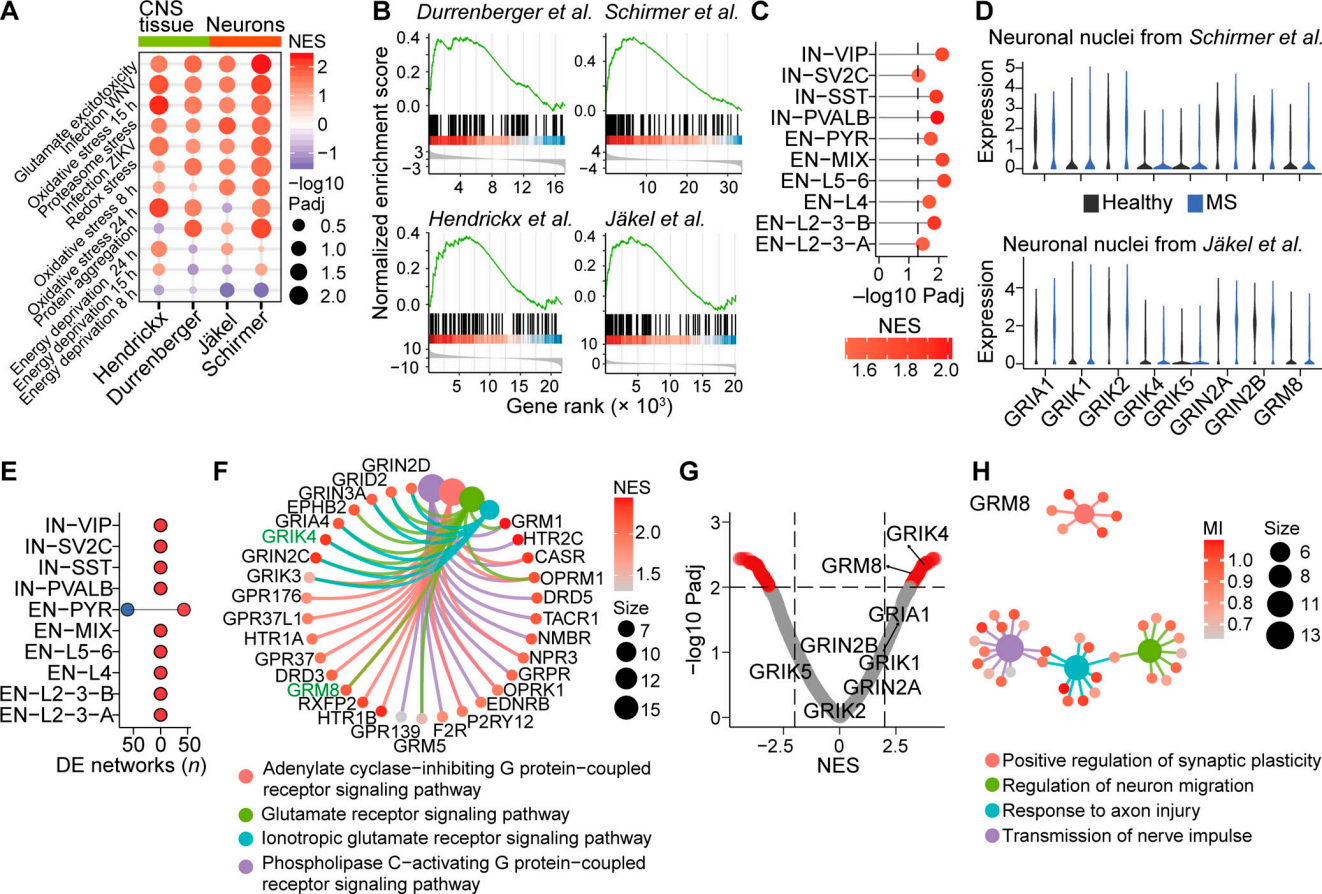
**Neuronal receptor interactomes in MS. (A)** GSEA of transcriptional signatures of defined neuronal stressors in MS CNS tissue or neurons. Rows are arranged in descending fashion by summed NESs across all MS datasets. **(B)** GSEA of glutamate stress signature in the respective MS transcriptomes. Transcriptomes in A and B were taken from Durrenberger et al. (2015), Hendrickx et al. (2017), Jäkel et al. (2019), and Schirmer et al. (2019). **(C)** Enrichment of glutamate stress signature in neuronal subsets from the cortices of MS patients. Dashed line represents significance threshold of FDR-adjusted P < 0.05. We classified subtypes provided by Schirmer et al. (2019) as inhibitory neurons (INs) that were defined by expression of vasoactive intestinal peptide (IN-VIP), synaptic vesicle glycoprotein 2C (IN-SV2C), somatostatin (IN-SST), parvalbumin (IN-PVALB), and excitatory neurons (ENs) from distinct layers (EN-L2-3A, -B, L4, L5-6), EN-PYRs, and a population without known identifier (EN-Mix). **(D)** Relative gene expression of MS-associated glutamate receptors in neuronal nuclei from MS patients. Transcriptomes were taken from Jäkel et al. (2019) and Schirmer et al. (2019). **(E)** The number of differentially regulated receptor interactomes with an FDR-adjusted P < 0.01 in neuronal subtypes from MS patients. **(F)** The top four significantly up-regulated biological themes and defining receptor interactomes in EN-PYRs. MS-associated glutamate receptors are labeled in green. **(G)** Volcano plot of receptor interactomes in EN-PYRs. Significantly enriched networks (FDR-adjusted P < 0.05) are labeled in red. MS-associated glutamate receptors are indicated. **(H)** Significantly enriched biological themes in the GRM8 regulatory network. Color represents mutual inference (MI), and size shows the number of genes in each respective biological theme. WNV, West Nile virus; ZIKV, Zika virus.

**Figure S1.**
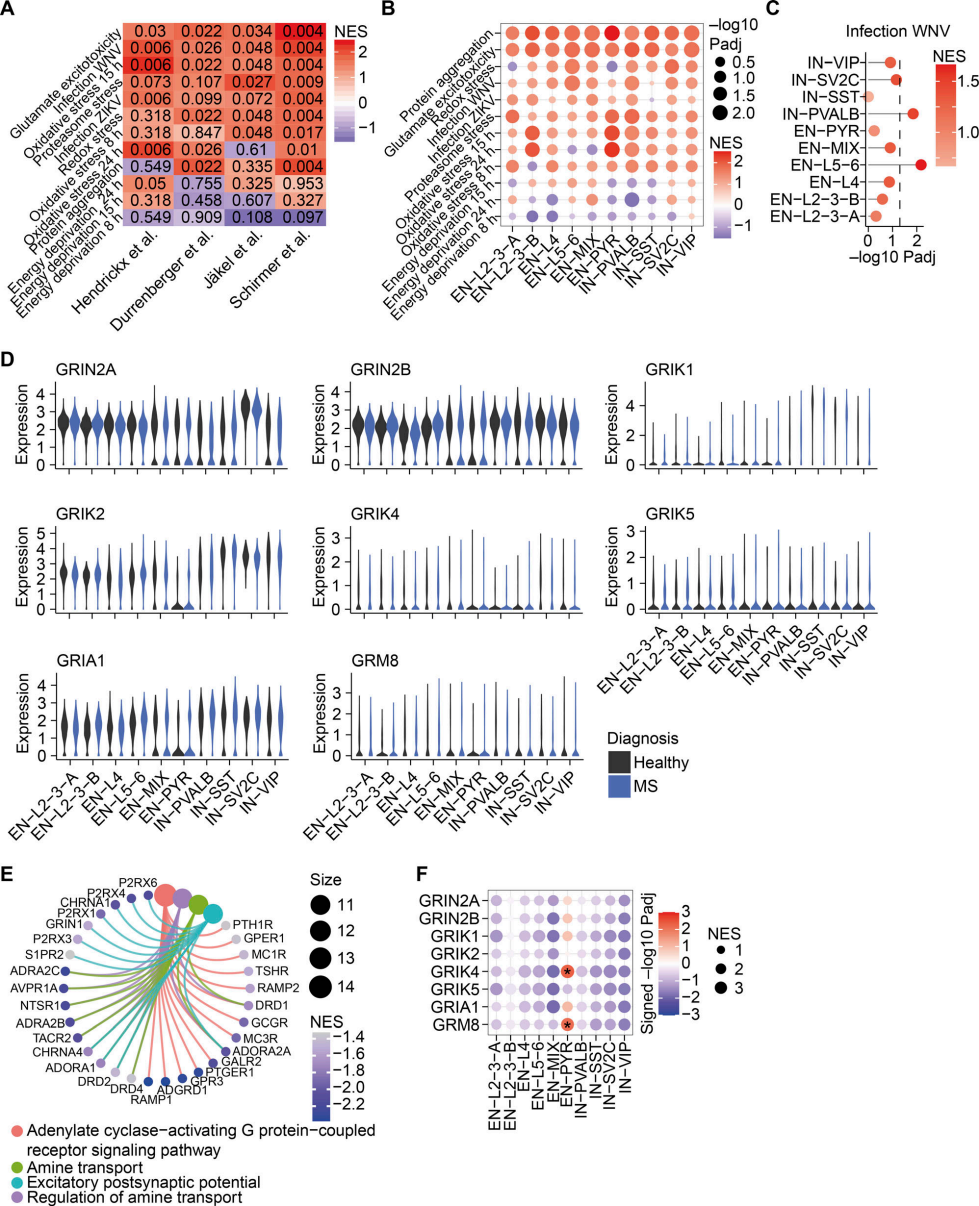
**Neuron-specific transcriptional stress signatures in MS. (A)** FDR-adjusted P values of the NESs of neuronal stress signature gene transcriptomes in respective MS brain specimens. Rows are sorted by cumulative NESs across MS transcriptomes. **(B)** Transcriptional enrichment of neuronal stress signature genes in respective neuron subtypes of MS brains from Schirmer et al. (2019). Size shows negative log_10_ FDR-adjusted P value; color represents NES. **(C)** Enrichment of gene signature genes from primary neurons that were transduced with West Nile virus (WNV) in depicted neuron subtypes of MS brains from Schirmer et al. (2019). **(D)** Relative gene expression of MS-associated *GRIN2A*, *GRIN2B*, *GRIK1*, *GRIK2*, *GRIK4*, *GRIK5*, *GRIA1*, and *GRM8* in different neuronal subtypes in brains of control and MS patients from Schirmer et al. (2019). **(E)** Top overrepresented biological themes in down-regulated receptor networks in pyramidal neurons of MS patients. **(F)** Heatmap of enrichment of MS-associated glutamate receptors in depicted neuron subtypes in MS patients from Schirmer et al. (2019). Significant enrichment with FDR-adjusted P < 0.01 is labeled with asterisks. Size represents NES. ZIKV, Zika virus.

To identify pathways that drive inflammation-induced neurodegeneration, we first compiled transcriptional signatures from primary neurons that were exposed to defined challenges, such as virally triggered inflammation (Daniels et al., 2017), glutamate excitotoxicity (Zhang et al., 2007), proteasomal inhibition (Choy et al., 2011), oxidative stress (Peng et al., 2012), protein aggregation (Kramer et al., 2018), or energy deprivation (Yap et al., 2013; signature genes are provided in Table S1). We then overlapped these signatures with bulk mRNA sequencing of MS gray (Durrenberger et al., 2015) and white matter lesions (Hendrickx et al., 2017), as well as neuronal transcriptomes derived from single-nucleus mRNA sequencing of MS cortices (Schirmer et al., 2019) and white matter (Jäkel et al., 2019) by gene set enrichment analysis (GSEA; Fig. 1 A). Notably, signature genes of glutamate excitotoxicity showed the highest enrichment across all MS datasets (Fig. 1, A and B; and Fig. S1 A), supporting that neuronal glutamate signaling is a major driver in MS neurodegeneration.

Due to the heterogeneity of neuronal populations, we next investigated stress responses in neuronal subtypes (Schirmer et al., 2019). We detected that the transcriptional signature of glutamate excitotoxicity (Fig. 1 C) as well as protein aggregation (Fig. S1 B) were significantly enriched in all subtypes, whereas inflammatory gene signatures were restricted to layer 5/6 excitatory neurons and parvalbumin-positive interneurons (Fig. S1 C). Hence, our results indicate that dysregulated glutamate signaling, together with protein aggregation, displays general pathological features of neurons that are chronically exposed to inflammation in MS, independent of subtype.

Previously, some glutamate receptor genes (*N*-methyl-D-aspartate [NMDA] receptor [NMDAR] subunits *GRIN2A*, *GRIN2B*; the kainate receptor subunits *GRIK1*, *GRIK2*, *GRIK4*, *GRIK5*; the α-amino-3-hydroxy-5-methyl-4-isoxazolepropionic acid [AMPA] receptor subunit *GRIA1*; and the metabotropic glutamate receptor *GRM8*) have been associated with MS disease severity (Baranzini et al., 2010, 2009; Strijbis et al., 2013; Wang et al., 2011). To investigate whether they contribute to our observed dysregulated glutamate signaling, we first compared their neuron-specific mRNA expression in control and MS patients (Schirmer et al., 2019; Jäkel et al., 2019), but we did not find any differences that could explain the disturbed glutamate signaling (Fig. 1 D and Fig. S1 D). Since the activity of transmembrane receptors heavily depends on mechanisms other than changes in mRNA expression, such as spatial organization, coincidental ligand binding, or desensitization (Strasser et al., 2017; Packiriswamy and Parameswaran, 2015), we next assessed the receptor activity by analyzing their downstream gene regulatory networks. Therefore, we employed the reconstruction of gene regulatory networks (ARACNe) reverse engineering algorithm (Margolin et al., 2006) and created neuron-specific receptor interactomes out of 502 available mRNA sequencing datasets (receptors are provided in Table S2; datasets are listed in Table S3) of healthy and stressed in vitro and in vivo mouse neuronal transcriptomes. Subsequently, we compared the obtained receptor networks (receptor interactomes are provided in Table S4) between distinct neuronal subtypes of MS patients and healthy controls. We found that excitatory pyramidal neurons (EN-PYRs) showed robust (*P*_adj_ < 0.01) down-regulation (*n* = 61) and up-regulation (*n* = 43) of transmembrane receptor networks (Fig. 1 E), which is consistent with their severe affliction in MS (Magliozzi et al., 2010). Notably, the regulatory networks of glutamate receptor signaling (Fig. 1 F and Fig. S1 E), and here in particular the MS-associated *GRIK4* and *GRM8* (Fig. 1 G and Fig. S1 F), were strongly enriched in EN-PYRs of MS patients. Intriguingly, the regulatory network of *GRM8* was enriched for genes that modulate neuroaxonal repair (Fig. 1 H). Therefore, we hypothesized that GRM8 activation could contribute to neuronal resilience during CNS inflammation and decided to mechanistically explore GRM8-dependent pathways in inflammation-induced glutamate excitotoxicity.

### Pre- and post-synaptic localization of GRM8 in neurons

**Figure S2.**
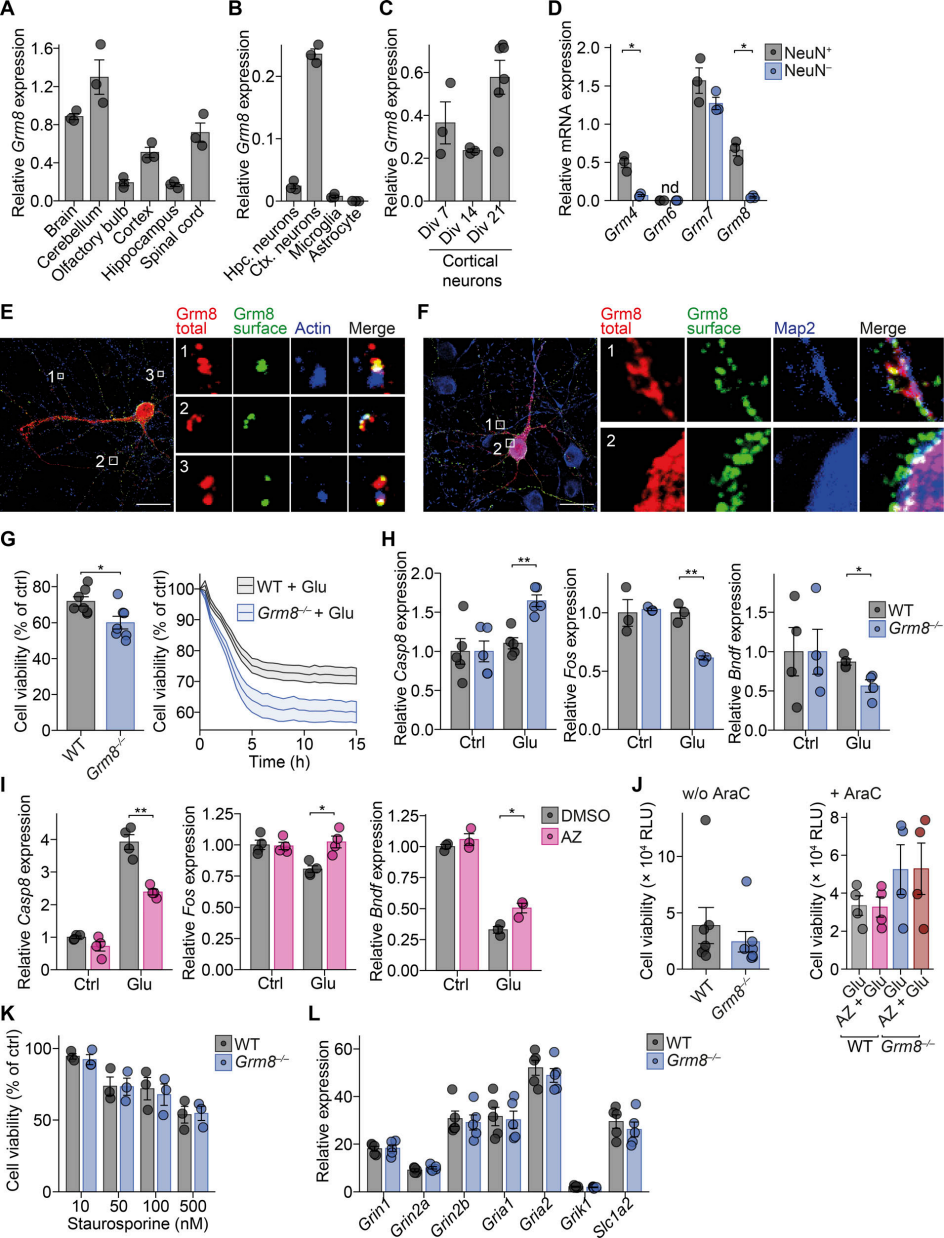
**Grm8 is located in close proximity to pre- and post-synapses and does not influence receptor expression. (A–C)** *Grm8* mRNA expression in indicated mouse tissue (A); hippocampal (hpc) neuronal, cortical (ctx) neuronal, and astrocyte cultures in vitro and sorted microglia (B); and in cortical neuronal cultures at 7, 14, and 21 div (C). All groups, *n* = 3. **(D)** mRNA expression of group 3 metabotropic glutamate receptors in sorted NeuN-positive and NeuN-negative nuclei from the spinal cords of healthy mice. All groups, *n* = 3. **(E and F)** Immunostaining of neuronal cultures that were transfected with EGFP-tagged *Grm8* cDNA and stained for surface and total EGFP and indicated proteins. Scale bars, 20 µm. **(G)** RealTime-Glo Cell Viability Assay of WT and *Grm8^−/−^* primary mouse neuronal cultures that were not depleted from glial cells and subjected to 20 µM glutamate for 15 h. All groups, *n* = 7. Data are normalized for each time point to the respective untreated neurons (Ctrl). **(H)** Relative mRNA expression of *Casp8* (left), *Fos* (middle), and *Bdnf* (right) in WT and *Grm8^−/−^* primary mouse neurons 16 div without glial cell depletion 4 h after application of 10 µM glutamate. Data were normalized to WT controls. *Casp8*, *n* = 5; *Fos*, *n* = 3; *Bdnf*, *n* = 4. **(I)** Relative mRNA expression of *Casp8* (left), *Fos* (middle), and *Bdnf* (right) in primary mouse neuronal cultures that were treated with 1 µM AZ for 24 h and were subsequently stimulated with 20 µM glutamate for 4 h. Data were normalized to DMSO-treated control. *Casp8*, *n* = 4; *Fos*, *n* = 4; *Bdnf*, *n* = 3. **(J)** RealTime-Glo Cell Viability Assay baseline relative luminescence units (RLU) without glial cell depletion of WT and *Grm8^−/−^* (without cytarabine [AraC]; left) and with glial cell depletion (+AraC; right); without AraC, *n* = 7; +AraC, *n* = 4. **(K)** RealTime-Glo Cell Viability Assay endpoint of WT and *Grm8^−/−^* primary mouse neurons 15 h after exposure to staurosporine in indicated concentrations. All groups, *n* = 3. **(L)** mRNA expression of indicated glutamate receptors in WT and *Grm8^−/−^* primary mouse neurons. All groups, *n* = 5. Data are shown as mean ± SEM. FDR-adjusted unpaired two-tailed *t* test was used with *, P < 0.05; **, P < 0.01.

**Figure 2.**
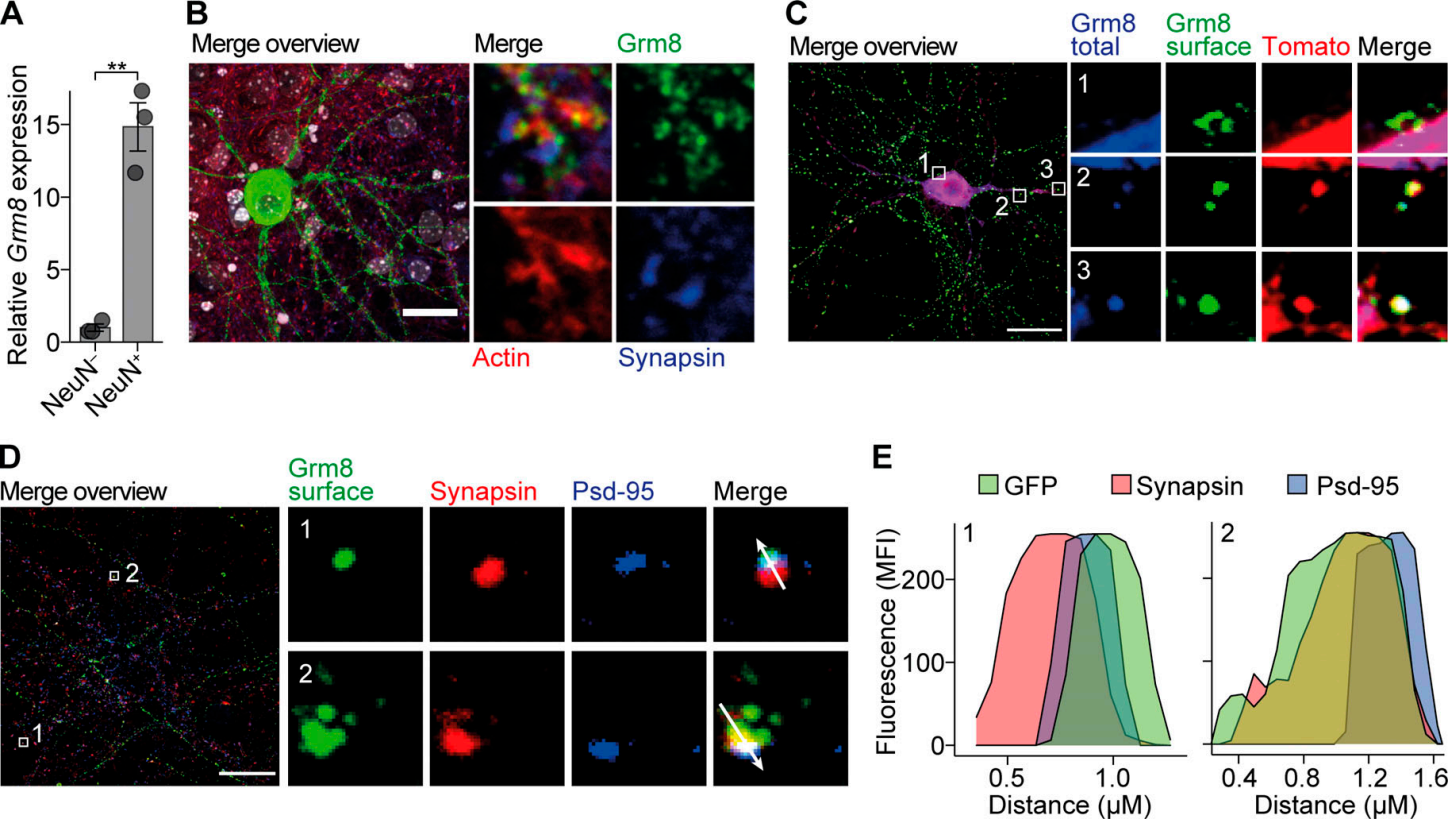
**Grm8 is located pre- and post-synaptically. (A)** *Grm8* expression in sorted NeuN-positive and NeuN-negative nuclei of the spinal cord. All groups, *n* = 3. Data are shown as mean ± SEM. FDR-adjusted unpaired two-tailed *t* test was used with **, P < 0.01. **(B–D)** EGFP was inserted at an extracellular domain of *Grm8* adjacent to its signal peptide (EGFP-*Grm8*). Neurons were transfected with EGFP-*Grm8* alone (B and D) or with EGFP-*Grm8* and a tdTomato-containing expression vector to visualize the entire neuronal morphology (C). Subsequently, living neurons were incubated with antibodies against EGFP at 4°C to visualize membrane-bound EGFP-*Grm8* (Grm8 surface), or antibodies against EGFP were applied to fixed and permeabilized neurons to visualize total EGFP-*Grm8* (Grm8 total). This was combined with immunostaining against the indicated markers of neuronal and synaptic morphology. Scale bars, 20 µm. **(E)** Histogram plots showing fluorescence intensity along the arrows of representative synapses from D of surface Grm8 (GFP), synapsin, and Psd-95.

GRM8 is an inhibitory mGluR that could potentially counteract glutamate excitotoxicity and confer neuroprotection in CNS inflammation. Since the function of GRM8 is poorly understood, we first characterized its CNS distribution and cellular localization in the mouse to get an indication of its contribution to neuronal responses during CNS inflammation. We observed strong *Grm8* mRNA expression in mouse cortex and spinal cord (Fig. S2, A–C). Moreover, we found expression of *Grm8* to be neuron specific, which was reflected by a 15-fold enrichment of *Grm8* in sorted mouse spinal cord NeuN-positive nuclei as compared with NeuN*-*negative nuclei (Fig. 2 A and Fig. S2 D). As existing antibodies raised against GRM8 showed unspecific staining (data not shown), we transfected primary mouse neuronal cultures with fluorescently tagged *Grm8*—enhanced GFP (EGFP) was inserted at the N-terminal extracellular domain adjacent to the signal peptide—to clarify the subcellular localization of GRM8. By applying antibodies directed against EGFP on living transfected neurons at 4°C to prevent receptor recycling, we were able to visualize surface-bound Grm8. Although previous antibody stainings reported presynaptic localization (Ferraguti and Shigemoto, 2006), in our transfected neurons, we observed a perisynaptic localization at neuronal somata and dendritic spines (Fig. 2, B–E; and Fig. S2, E and F). This close proximity to neighboring excitatory glutamate receptors might allow GRM8 to efficiently modulate glutamate-induced excitotoxicity in neurons.

### GRM8 activation is neuroprotective by suppressing ER calcium release

**Figure 3.**
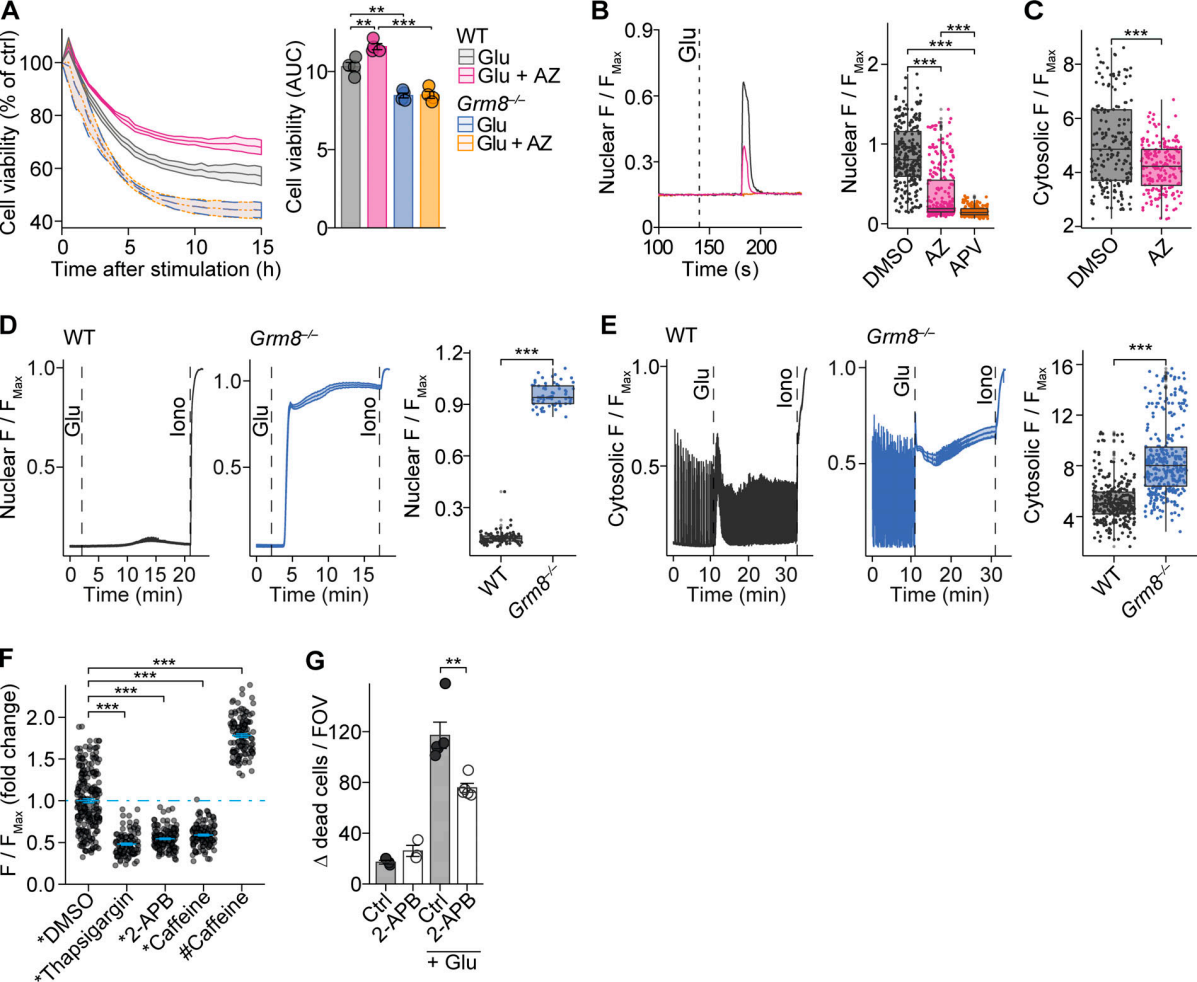
**Grm8 protects against glutamate-induced calcium accumulation. (A)** RealTime-Glo Cell Viability Assay of WT and *Grm8^−/−^* mouse neurons ± AZ pretreatment that were exposed to glutamate. All groups, *n* = 4. **(B and C)** Nuclear (B; DMSO, *n* = 247; AZ, *n* = 269; APV, *n* = 185) and cytosolic (C; DMSO, *n* = 227; AZ, *n* = 213) calcium recordings in glutamate-exposed mouse neurons that were pretreated with AZ. Data are shown as median ± interquartile range. **(D and E)** Nuclear (D; WT, *n* = 91; *Grm8^−/−^*, *n* = 64) and cytosolic (E; WT, *n* = 298; *Grm8^−/−^*, *n* = 324) calcium recordings in glutamate-exposed WT and *Grm8^−/−^* mouse neurons. Data are shown as median ± interquartile range. **(F)** Mouse neuronal calcium levels after emptying the ER (pretreatment with thapsigargin or 2-APB or caffeine) or enhancing ER release probability (caffeine) with subsequent (*) or concurrent (#) glutamate exposure. Data are normalized to glutamate-induced calcium increase after DMSO pretreatment. DMSO, *n* = 231; thapsigargin, *n* = 105; 2-APB, *n* = 145; caffeine pretreatment, *n* = 134; caffeine concurrent treatment, *n* = 123. **(G)** Mouse neuronal cultures were exposed to glutamate ± pretreatment with 20 µM 2-APB, and dead cells were counted. Ctrl, *n* = 3; 2-APB, *n* = 3; Glu + Ctrl, *n* = 5; Glu + 2-APB, *n* = 5. If not stated otherwise, data are shown as mean ± SEM. FDR-adjusted unpaired two-tailed *t* test was used with **, P < 0.01; ***, P < 0.001. FOV, field of view.

**Figure S3.**
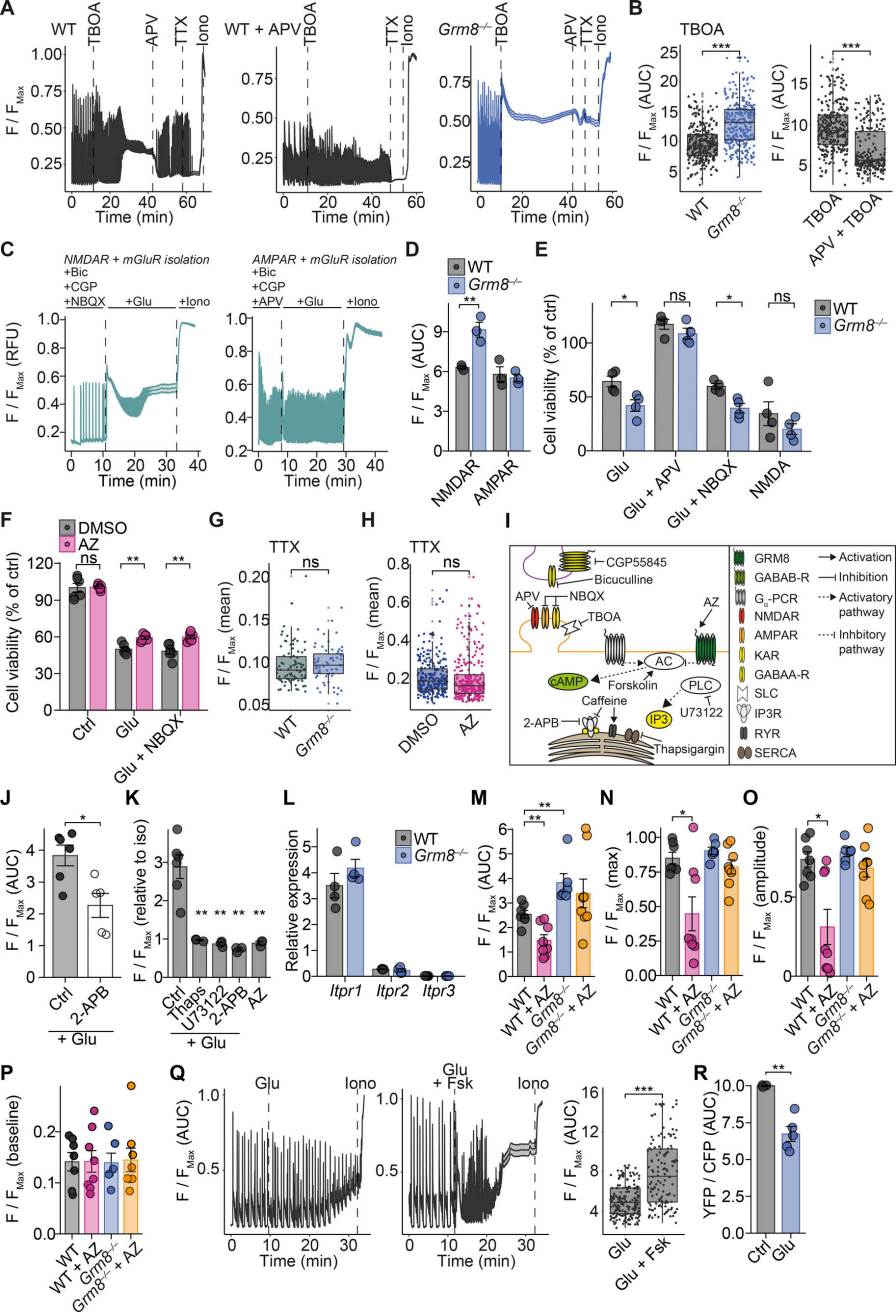
***Grm8*-deficient neurons show stronger glutamate-induced calcium accumulation. (A)** Representative calcium traces of WT (left) and *Grm8^−/−^* (right) neuronal cultures without glial cell depletion that were sequentially challenged with 50 µM TBOA, 50 µM APV (only left and right panel), 1 µM TTX, 8 µM ionomycin, and WT neuronal cultures that were similarly treated but additionally incubated with 50 µM APV during TBOA challenge (middle). **(B)** Left, quantification of AUC of cytosolic calcium in WT (*n* = 307) and *Grm8^−/−^* (*n* = 314) neurons that were challenged with 50 µM TBOA for 30 min. WT, *n* = 307; *Grm8^−/−^*, *n* = 314. Right, quantification of neuronal cultures that were challenged with 50 µM TBOA ± 50 µM APV at the same time for 30 min. TBOA, *n* = 296; APV + TBOA, *n* = 233. **(C and D)** Isolated mGluR and NMDAR (left) or AMPA receptor (AMPAR; right) activation in mouse WT and *Grm8^−/−^* neurons. All groups, *n* = 3. **(E)** RealTime-Glo Cell Viability Assay endpoint of WT and *Grm8^−/−^* primary neurons that were exposed to 20 µM glutamate, 20 µM glutamate and 50 µM APV, 20 µM glutamate and 10 µM NBQX, and NMDA. All groups, *n* = 4. **(F)** CellTiter-Glo Viability Assay of primary mouse neurons that were treated with 0.1% DMSO or 1 µM AZ for 24 h and were subsequently exposed to control conditions, 20 µM glutamate, 20 µM glutamate, and 10 µM NBQX for 15 h. Data were normalized to DMSO-treated controls. All groups, *n* = 6. **(G and H)** Mean baseline calcium level of WT (*n* = 91) and *Grm8^−/−^* (*n* = 64) silenced neurons (G; WT, *n* = 91; *Grm8^−/−^*, *n* = 64) and DMSO- and AZ-treated neurons (H; DMSO, *n* = 247; AZ, *n* = 269). **(I)** Graphical summary of chemicals and their respective targets and functions used for experiments. **(J)** Calcium response to glutamate of mGluR- and NMDAR-isolated mouse neuronal cultures that were treated with 0.1% DMSO or 50 µM 2-APB. Ctrl, *n* = 6; 2-APB, *n* = 5. **(K)** Mouse neuronal cultures were subjected to mGluR isolation protocol and were additionally incubated with 1 µM thapsigargin, 1.25 µM U73122, and 50 µM 2-APB for 10 min and subsequently with 20 µM glutamate or 1 µM AZ without glutamate. Ctrl, *n* = 7; other conditions, *n* = 3. Data were normalized to mean calcium level during isolation before application of glutamate. **(L)** Relative mRNA expression of IP3R paralogs *Itpr1*, *Itpr2*, and *Itpr3* in WT and *Grm8^−/−^* neuronal cultures. All groups, *n* = 4. **(M–P)** WT and *Grm8^−/−^* neuronal cultures were treated with 0.1% DMSO or 1 µM AZ, and subsequently isolated mGluRs were activated with glutamate. Quantification of AUC (M), maximal response (N), maximal amplitude (O), and mean baseline during mGluR isolation (P) is shown. WT, *n* = 7; *Grm8^−/−^*, *n* = 6; WT + AZ, *n* = 7; *Grm8^−/−^* + AZ, *n* = 8. **(Q)** Spontaneously active cultures were exposed to 20 µM glutamate or 20 µM glutamate together with 10 µM forskolin (Fsk). Data are shown as median ± SEM. Glu, *n* = 184; Glu + Fsk, *n* = 132. **(R)** CFP/YFP ratios that negatively correlate with cAMP of mGluR-isolated neuronal cultures that were subsequently vehicle treated or 20 µM glutamate treated. Ctrl, *n* = 3; Glu, *n* = 5. Data are shown as mean ± SEM. FDR-adjusted unpaired two-tailed *t* test was used with *, P < 0.05; **, P < 0.01; ***, P < 0.001.

Next, we investigated the potential of GRM8 to modulate glutamate-mediated neuronal loss. We compared glutamate-challenged *Grm8*-deficient (Duvoisin et al., 2005) with WT primary mouse neurons that were pretreated with a positive allosteric modulator of GRM8 AZ12216052 (AZ; Jantas et al., 2014; Rossi et al., 2014) or vehicle control. Following glutamate exposure, there was increased injury of *Grm8^−/−^* neurons compared with WT neurons (Fig. 3 A and Fig. S2 G) that was accompanied by transcript induction of proapoptotic caspase-8 (*Casp8*) and repression of the prosurvival genes FBJ osteosarcoma oncogene (*Fos*) and brain-derived neurotrophic factor (*Bdnf*; Fig. S2, H and I). Moreover, pharmacological activation of Grm8 by AZ rescued WT but not *Grm8^−/−^* neurons from glutamate excitotoxicity (Fig. 3 A). We observed no differences in baseline viability, apoptotic potential, and glutamate receptor expression between WT and *Grm8^−/−^* neurons (Fig. S2, J–L). Since cytosolic and nuclear calcium accumulation has been proposed to drive glutamate excitotoxicity (Lau and Tymianski, 2010), we next analyzed whether GRM8-mediated modulation of neuronal calcium levels could explain its protection against neuronal hyperexcitation. Application of glutamate to spontaneously active neurons or electrically silenced neurons resulted in an NMDAR-dependent nuclear calcium accumulation over time (Fig. 3 B). However, neuronal activation of Grm8 with AZ resulted in reduced nuclear and cytosolic calcium accumulation compared with vehicle treatment (Fig. 3, B and C). Accordingly, *Grm8^−/−^* neurons showed an exaggerated nuclear and cytosolic calcium accumulation (Fig. 3, D and E). Similarly, blocking synaptic glutamate reuptake (Fig. S3, A and B) or specifically triggering NMDAR and mGluR activity (Fig. S3, C and D) resulted in enhanced calcium accumulations and cell death (Fig. S3, E and F) in *Grm8*-deficient neurons compared with WT neurons. *Grm8* deficiency or activation by AZ did not change neuronal baseline calcium level (Fig. S3, G and H). Thus, GRM8 activation is neuroprotective by reducing glutamate-induced calcium accumulation.

The ER constitutes the major intracellular calcium store, which can be released upon stimulation. To explore its contribution to glutamate toxicity, we emptied the ER calcium store by pretreatment with thapsigargin or caffeine, both of which resulted in reduced glutamate-induced calcium accumulation (Fig. 3 F). Similarly, inhibition of ER calcium release by blocking IP3R with 2-APB led to reduced calcium accumulation and ameliorated glutamate-induced neuronal injury (Fig. 3, F and G; and Fig. S3, I and J). Of note, simultaneous application of glutamate and caffeine that increases the ER calcium release probability further increased the glutamate-induced calcium response (Fig. 3 F). Together, this supports the notion that calcium release from the ER and iGluR-mediated external calcium entry synergistically drive glutamate excitotoxicity.

### GRM8 inhibits ER-mediated calcium release via cAMP and IP3R signaling

**Figure 4.**
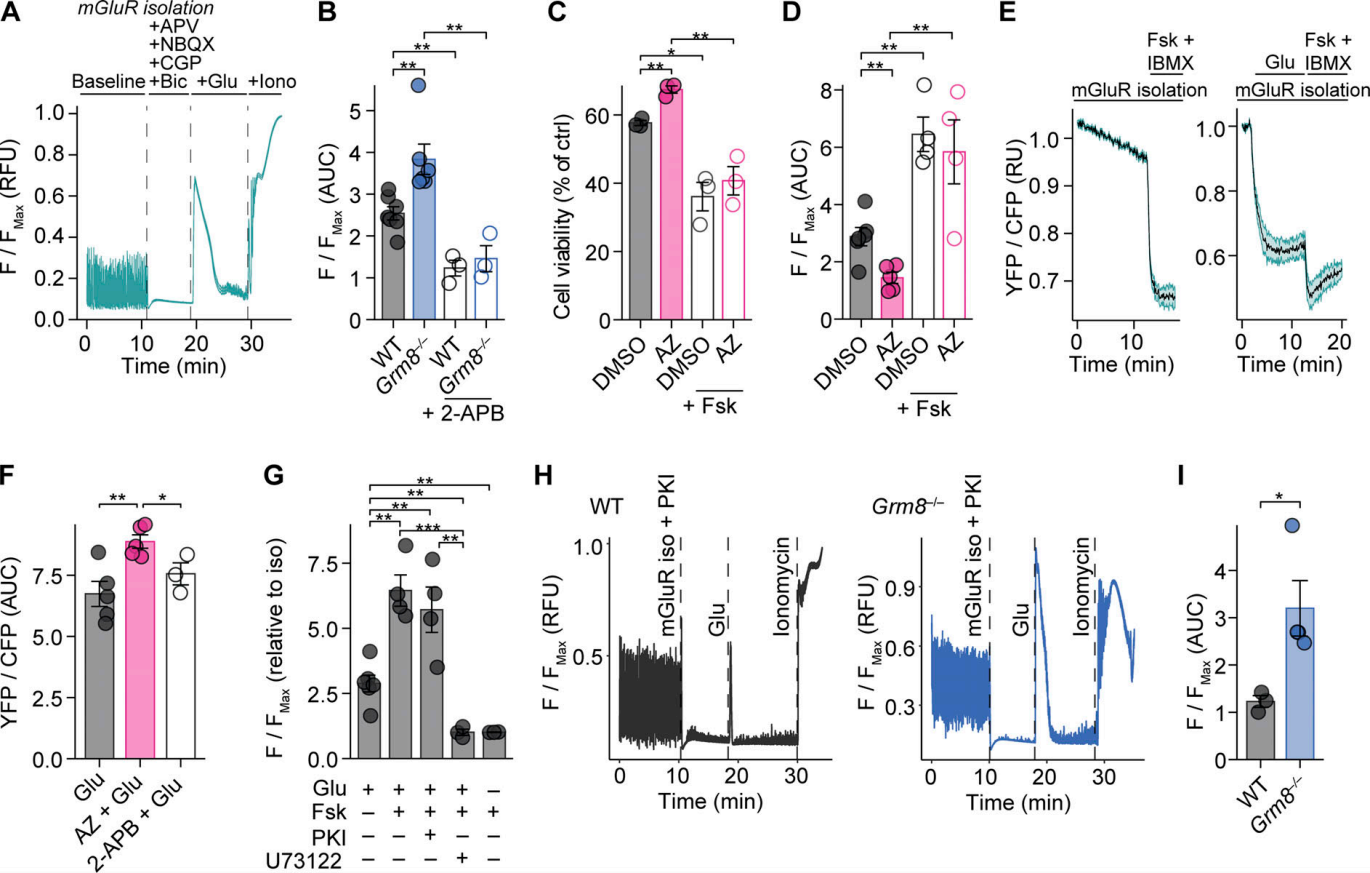
**Grm8 inhibits IP3R-dependent calcium release. (A and B)** Isolated mGluR calcium response (A) in WT and *Grm8^−/−^* mouse neurons with or without pretreatment with 2-APB (B; WT, *n* = 7; *Grm8^−/−^*, *n* = 6; WT + 2-APB, *n* = 3; *Grm8^−/−^* + 2-APB, *n* = 3). **(C and D)** Cell viability (C; all groups, *n* = 3) and isolated mGluR calcium response (D; DMSO, *n* = 6; AZ, *n* = 5; DMSO + Fsk, *n* = 3; AZ + Fsk, *n* = 3) after AZ with or without forskolin (Fsk) pretreatment and subsequent glutamate application. **(E and F)** Mouse neuronal cAMP response during isolated mGluR activation (E) and AZ or 2-APB pretreatment (F; Glu, *n* = 5; Glu + AZ, *n* = 5; 2-APB + Glu, *n* = 3). **(G)** Isolated mGluR calcium response in WT neurons that were additionally treated with forskolin, PKI, or PLC inhibitor (U73122) in the indicated combinations (Glu, *n* = 6; Glu + Fsk, Glu + Fsk + PKI, *n* = 4; Glu + Fsk + U73122, Fsk, *n* = 3). **(H and I)** Representative calcium traces (H) and quantification (I) of isolated mGluR calcium response from WT (*n* = 3) and *Grm8^−/−^* (*n* = 4) primary neurons that were additionally pretreated with PKI. For quantification of calcium and cAMP, AUC was used; if not stated otherwise, data are shown as mean ± SEM. FDR-adjusted unpaired two-tailed *t* test was used with *, P < 0.05; **, P < 0.01; ***, P < 0.001. RFU, relative fluorescence units.

As GRM8 has been reported to reduce excitatory synaptic transmission (Rossi et al., 2014; Gosnell et al., 2011), we hypothesized that its effect to counteract glutamate excitotoxicity is mediated by inhibiting calcium release from the ER (Chen-Engerer et al., 2019). To test this hypothesis, we pharmacologically isolated mGluR-specific calcium responses (Fig. 4 A) that were dependent on sarco/ER calcium–ATPase, IP3R, and phospholipase C (PLC) activation (Fig. S3 K). *Grm8*-deficient primary neurons showed enhanced mGluR-mediated calcium release from the ER that was abolished by inhibiting IP3R activity (Fig. 4 B). Of note, *Grm8* deficiency did not affect neuronal IP3R expression (*Itpr1–3*; Fig. S3 L). Congruently, AZ pretreatment reduced the mGluR-dependent cytosolic calcium increase in WT but not *Grm8^−/−^* primary neurons (Fig. S3, M–P). We concluded that Grm8 activation desensitizes IP3R-mediated calcium release from the ER.

Since GRM8 activation has been shown to increase Gα_i_ activity (Duvoisin et al., 2010), we reasoned that decreasing cytosolic levels of cAMP is responsible for restricting IP3R-evoked calcium release (Taylor, 2017). Therefore, we investigated whether pharmacological increase of cAMP affects glutamate excitotoxicity. We observed that forskolin-mediated acute increase of cAMP synergistically enhanced glutamate-mediated calcium accumulation (Fig. S3 Q) and cell death (Fig. 4 C). More specifically, cytosolic cAMP accumulation enhanced mGluR-dependent calcium release from the ER, overriding the protective effect of AZ (Fig. 4 D). To directly verify that stimulatory mGluR activation increases cAMP that is counteracted by GRM8 activity, we used primary neurons derived from a transgenic fluorescence resonance energy transfer (FRET)–based cAMP biosensor mouse (Börner et al., 2011). Isolated mGluR activation resulted in an increase of intracellular cAMP (Fig. 4 E and Fig. S3 R). Moreover, pretreatment with AZ, but not blocking IP3R, reduced the glutamate-induced increase of cAMP, indicating that GRM8 counteracts glutamate-induced cAMP production upstream of the IP3R (Fig. 4 F). Thus, GRM8 protects from glutamate-induced neurotoxicity by limiting cAMP-mediated IP3R sensitization that reduces calcium release from the ER.

Next, we thought that the cAMP-induced IP3R sensitization could be mediated by activation of protein kinase A (PKA). Notably, we found that pretreatment of primary neurons with the PKA inhibitor (PKI; 5-24) did not limit the enhancing effect of forskolin on the mGluR-specific calcium response. By contrast, isolated mGluR and forskolin-enhanced mGluR calcium response could be completely abolished by treating cells with the PLC inhibitor U73122 (Fig. 4 G). Thus, cAMP accumulation sensitizes IP3Rs and thereby increases calcium release from the ER independent of PKA activity. Accordingly, treatment of *Grm8^−/−^* primary neurons with PKI did not rebalance the isolated mGluR calcium response (Fig. 4, H and I). Together, glutamate engagement of neuronal activatory mGluRs results in cAMP accumulation that directly sensitizes IP3Rs and hence controls cytosolic calcium levels and cell death, which is limited by GRM8 activity.

### GRM8 activation as a neuroprotective strategy in CNS inflammation in vivo

**Figure 5.**
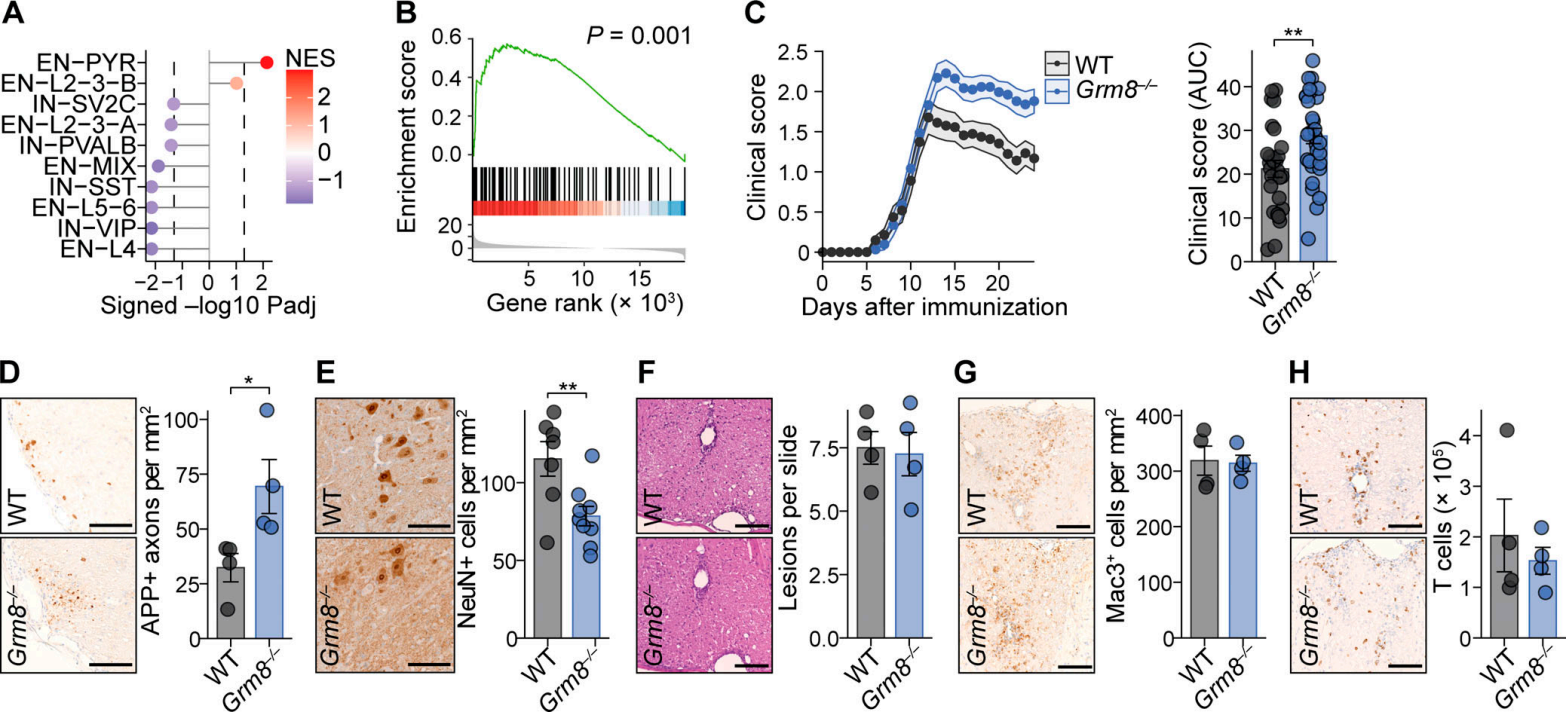
***Grm8* deficiency aggravates neurodegeneration and clinical disability in EAE. (A)** Transcriptional enrichment of human cortical neuron subtype–defining genes from Schirmer et al. (2019) and mouse spinal cord ChAT-positive motor neurons from Schattling et al. (2019). Dashed lines represent the significance threshold of FDR-adjusted P < 0.01. **(B)** GSEA of transcriptional glutamate stress signature in ranked gene list from Schattling et al. (2019; NES, 0.573). **(C)** WT (*n* = 27) and *Grm8^−/−^* (*n* = 31) mice were subjected to EAE. Pooled data from three independent experiments are shown. AUC was quantified. WT, *n* = 27; *Grm8^−/−^*, *n* = 31. **(D and E)** Histopathological quantification of damaged APP-positive axons during acute inflammation 15 d after immunization (D; all groups, *n* = 4) and neuronal loss in the chronic phase 30 d after immunization (E; WT, *n* = 9; *Grm8^−/−^*, *n* = 7) of WT and *Grm8^−/−^* EAE mice. **(F–H)** Histopathological quantification of inflammatory lesions (F) and Mac3-positive cells (G) and FACS quantification of T cell infiltration (H) during acute phase of EAE 15 d after immunization. All groups, *n* = 4. Scale bars, 100 µm. Data are shown as mean values ± SEM. FDR-adjusted Mann–Whitney *U* test was used with *, P < 0.05; **, P < 0.01.

**Figure S4.**
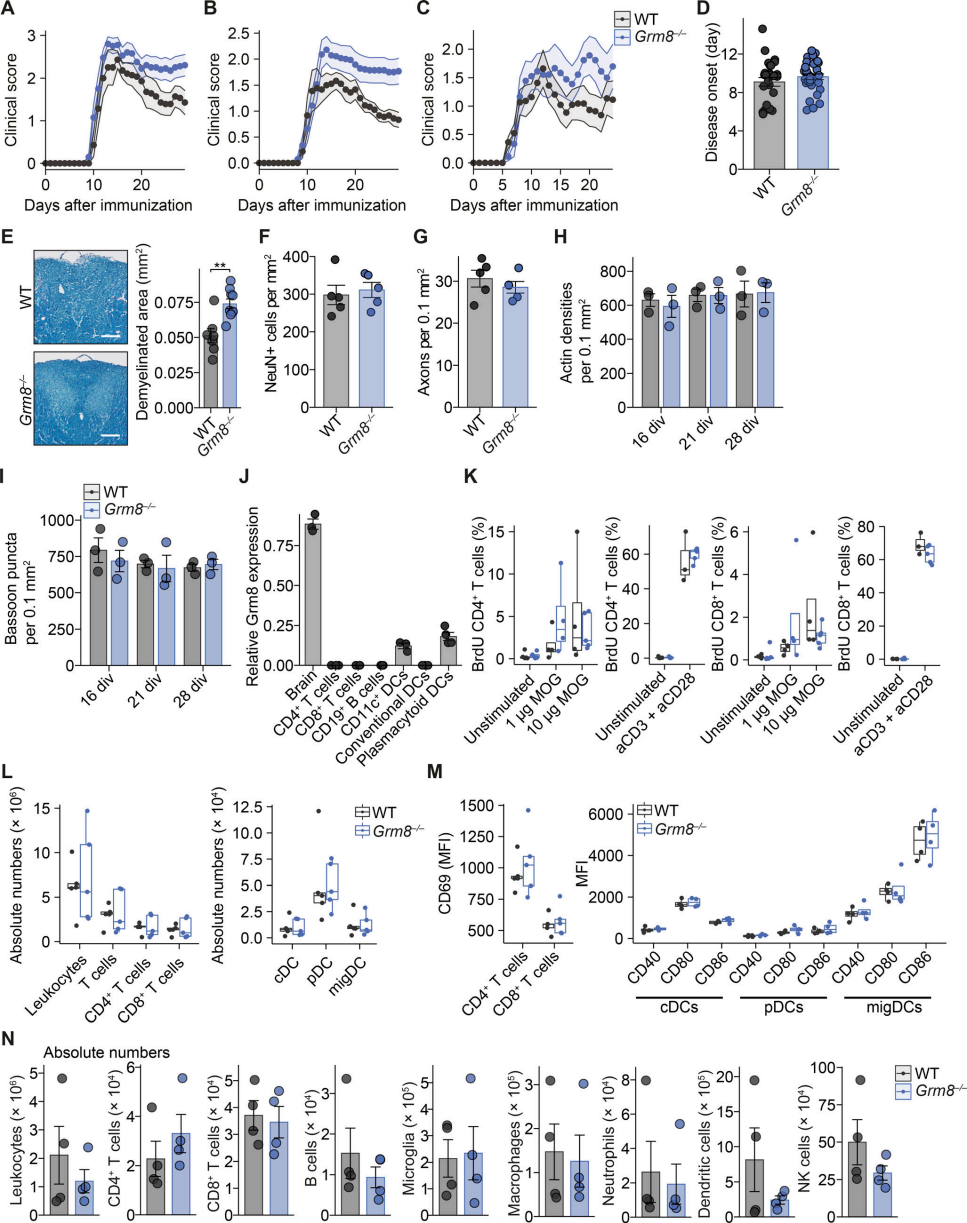
***Grm8* deficiency does not alter baseline axonal and syaptic density or immune response in EAE. (A–C)** Disease course of individual EAEs that are shown as pooled data in Fig. 5 C. In A, WT, *n* = 7; *Grm8^−/−^*, *n* = 10. In B, WT, *n* = 9; *Grm8^−/−^*, *n* = 13. In C, WT, *n* = 11; *Grm8^−/−^*, *n* = 9. Statistics are provided in Table S5. **(D)** Day of disease onset of WT and *Grm8^−/−^* animals that were subjected to EAE. WT, *n* = 27; *Grm8^−/−^*, *n* = 31. **(E)** Quantification of demyelinated area by Luxol blue staining in dorsal columns of spinal cords from WT (*n* = 7) and *Grm8^−/−^* (*n* = 9) mice in the chronic phase of EAE 30 d after immunization. **(F and G)** Number of neurons (F) and axons (G) in spinal cords of healthy WT and *Grm8^−/−^* mice. All groups, *n* = 5. **(H and I)** Actin densities (H) and bassoon puncta (I) of WT and *Grm8^−/−^* neuronal cultures at indicated div. **(J)** Relative *Grm8* mRNA expression in the mouse brain and in indicated immune cell subsets. All groups, *n* = 3. **(K)** Quantification of BrdU-positive T cells that were derived from draining lymph nodes 9 d after immunization and were restimulated with MOG_35–55_ or CD3/CD28 antibodies as a positive control and pulsed with BrdU for 16 h. WT unstimulated, *n* = 5; 1 µg of MOG, *n* = 4; 10 µg of MOG *n* = 4; aCD3 + aCD28, *n* = 3; *Grm8^−/−^*, unstimulated, *n* = 5; 1 µg of MOG *n* = 4; 10 µg of MOG, *n* = 4; aCD3 + aCD28, *n* = 5. **(L)** Quantification of immune cell populations that were derived from draining lymph nodes 9 d after immunization. WT, *n* = 5; *Grm8^−/−^*, *n* = 5. **(M)** Quantification of MFI of the activation marker CD69 in T cells and activation markers CD40, CD80, and CD86 in depicted dendritic cell populations that were derived from draining lymph nodes 9 d after immunization of WT and *Grm8^−/−^* mice. For CD69, all groups, *n* = 4; for CD40, CD80, and CD86, all groups, *n* = 4. **(N)** Quantification of absolute numbers of CNS-infiltrating immune cell populations per spinal cord of WT and *Grm8^−/−^* mice during the acute phase of EAE 15 d after immunization. All groups, *n* = 4. Data are shown as mean ± SEM. FDR-adjusted unpaired two-tailed *t* test was used with **, P < 0.01. cDC, conventional dendritic cell; pDC, plasmacytoid dendritic cell; migDC, migratory dendritic cell.

To examine whether our in vitro findings could be translated into in vivo models of CNS inflammation, we investigated the neuroprotective potential of GRM8 activation in the MS mouse model of EAE. Since inflammation in C57BL/6 EAE mice strongly affects motor neurons in the mouse spinal cords, we first probed whether mouse motor neurons show a transcriptional similarity to layer 5 pyramidal neurons from human cortices, which was indeed the case (Fig. 5 A). Moreover, similar to MS pathology, motor neurons from EAE animals (Schattling et al., 2019) showed enrichment of gene transcripts that are indicative of glutamate excitotoxicity (Fig. 5 B). As these overlapping key characteristics support translatability of neuronal responses in mice to humans during CNS inflammation, we compared *Grm8^−/−^* and WT animals that were subjected to EAE. In accordance with our in vitro findings, *Grm8^−/−^* mice showed an exacerbated EAE disease course compared with WT animals, especially in the chronic phase of EAE (pooled data from three independent experiments are shown in Fig. 5 C; results from individual EAE experiments are provided in Fig. S4, A–C, and Table S5), while disease onset was unaltered (Fig. S4 D). There was an increased number of amyloid precursor protein (APP)–positive axons, a marker for axonal injury, in the acute phase of EAE (Fig. 5 D) and an extensive loss of neurons in the ventral horn of the spinal cord (Fig. 5 E) and demyelination in the dorsal columns (Fig. S4 E) of the spinal cord in the chronic phase of EAE. Importantly, healthy WT and *Grm8^−/−^* mice did not differ in axonal and neuronal counts in vivo and synaptic density in vitro (Fig. S4, F–I). As we detected *Grm8* expression in plasmacytoid dendritic cells but not in other immune cell subsets (Fig. S4 J), we examined whether the ameliorated neuronal loss in *Grm8^−/−^* mice could be partly explained by altered immune cell activation or infiltration. However, genetic deletion of *Grm8* neither affected the proliferation of MOG_35–55_-specific T cells by recall stimulation ex vivo (Fig. S4, K) nor impacted on frequencies, absolute numbers, or activation of dendritic cell subsets or T cells during disease onset (Fig. S4, L and M). Moreover, the numbers of inflammatory lesions and infiltrating immune cells were not altered in *Grm8^−/−^* mice compared with WT mice during the acute phase of EAE (Fig. 5, F–H; and Fig. S4 N). Together, *Grm8* deficiency results exclusively in higher neuronal vulnerability to inflammation-induced neurodegeneration.

**Figure S5.**
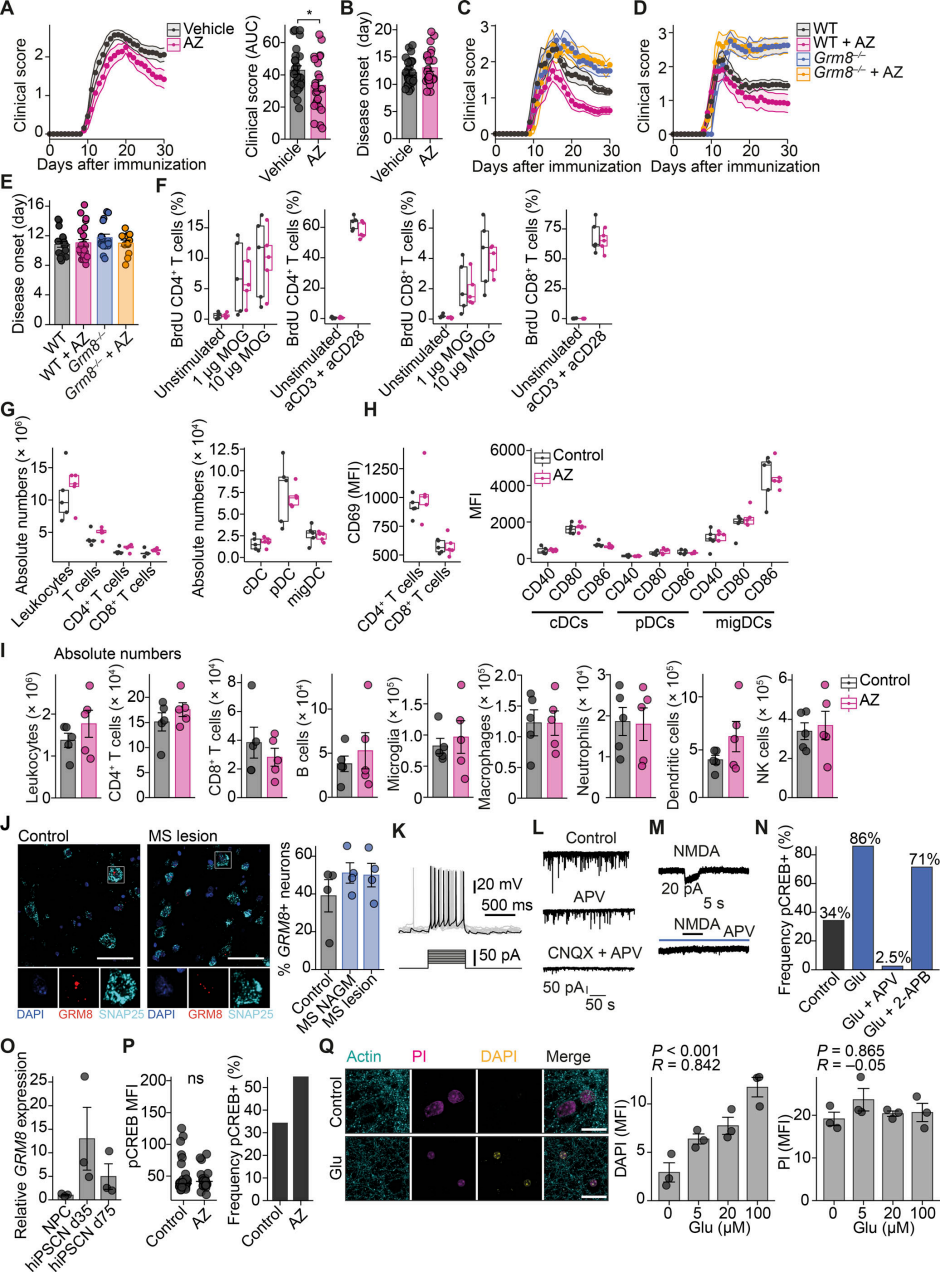
**Assessment of the immune response in EAE and hiPSC physiology after pharmacological Grm8 activation. (A and B)** Animals were subjected to EAE and were injected with either vehicle or AZ. AUC (A) and day of disease onset (B) were quantified. Vehicle, *n* = 26; AZ, *n* = 23. FDR-adjusted Mann–Whitney *U* test was used. **(C and D)** Disease course of individual EAEs that are shown as pooled data in Fig. 6 A. In C, WT, *n* = 10; *Grm8^−/−^*, *n* = 8; WT + AZ, *n* = 12; *Grm8^−/−^* + AZ, *n* = 6. In D, WT, *n* = 8; *Grm8^−/−^*, *n* = 8; WT + AZ, *n* = 11; *Grm8^−/−^* + AZ, *n* = 6. Statistics are provided in Table S5. **(E)** Day of disease onset of WT and *Grm8^−/−^* animals that were injected i.p. with a vehicle or AZ. WT, *n* = 18; *Grm8^−/−^*, *n* = 17; WT + AZ, *n* = 23; *Grm8^−/−^* + AZ, *n* = 12. FDR-adjusted Mann–Whitney *U* test was used. **(F)** Quantification of BrdU-positive T cells that were derived from draining lymph nodes 9 d after immunization of mice that were treated for 6 d with DMSO vehicle (control) or AZ and were restimulated with MOG_35–55_ or CD3/CD28 as a positive control and pulsed with BrdU for 16 h. Control, *n* = 5; AZ, *n* = 5. **(G)** Quantification of immune cell populations that were derived from draining lymph nodes 9 d after immunization of mice that were treated for 6 d with DMSO vehicle (control) or AZ. Control, *n* = 5; AZ, *n* = 5. **(H)** Quantification of MFI of the activation marker CD69 in T cells and activation markers CD40, CD80, and CD86 in depicted dendritic cell populations that were derived from draining lymph nodes 9 d after immunization of mice that were treated for 6 d with DMSO vehicle (control) or AZ. Control, *n* = 5; AZ, *n* = 5. **(I)** Quantification of absolute numbers of CNS-infiltrating immune cell populations per spinal cord of mice that were treated with either vehicle or AZ during the acute phase of EAE 15 d after immunization. All groups, *n* = 5. **(J)** RNAscope fluorescence in situ hybridization of *GRM8* transcripts in brain sections of control individuals and MS NAGM and cortical lesions. All groups, *n* = 4. Scale bars, 50 µm. **(K)** In current clamp, stepwise increase of current injections in hiPSC neurons results in depolarization and neuronal firing. **(L)** In voltage clamp at −70 mV, subsequent application of APV and CNQX reduces spontaneous excitatory post-synaptic currents in hiPSC neurons. **(M)** Application of 50 µM NMDA for 4 s to hiPSC neurons in the presence of 0.5 µM TTX, 20 µM bicuculline, and 20 µM CNQX induces inward currents at a holding potential of −70 mV (17.4 ± 2.9 pA; *n* = 5) that can be completely blocked by 50 µM APV. **(N)** Frequency of pCREB-positive hiPSC neurons after stimulation with 20 µM glutamate, 20 µM glutamate with 50 µM APV, or 20 µM glutamate with 50 µM 2-APB for 20 min. Control, *n* = 38; Glu, *n* = 50; Glu + APV, *n* = 78; Glu + 2-APB, *n* = 49. **(O)** Relative mRNA expression of *GRM8* in undifferentiated human NPCs and 35 or 75 d after differentiation into hiPSC neurons. All groups, *n* = 3. **(P)** hiPSC neurons were treated for 20 min with either 0.1% DMSO or 1 µM AZ (pCREB-positive neurons; control, 54%; AZ, 34%). Control, *n* = 38; AZ, *n* = 22. **(Q)** Neuronal cultures were stimulated with indicated concentrations of glutamate for 2 h, and subsequently 5 µM DAPI was added for 15 min (yellow). After permeabilization, PI (magenta) was used to stain all nuclei and actin (cyan) to visualize neuronal morphology. Left, representative image of vehicle-treated (control) and 20 µM glutamate–stimulated cultures after 2 h. Middle, quantification of nuclear DAPI fluorescence after exposure to indicated glutamate concentration (*R* = 0.842). Right, quantification of nuclear PI fluorescence after exposure to indicated glutamate concentrations (*R* = −0.05). All groups, *n* = 3. Pearson correlation was used. Data are shown as mean ± SEM. Scale bars, 20 µm. FDR-adjusted unpaired two-tailed *t* test was used with *, P < 0.05. cDC, conventional dendritic cell; pDC, plasmacytoid dendritic cell; migDC, migratory dendritic cell.

**Figure 6.**
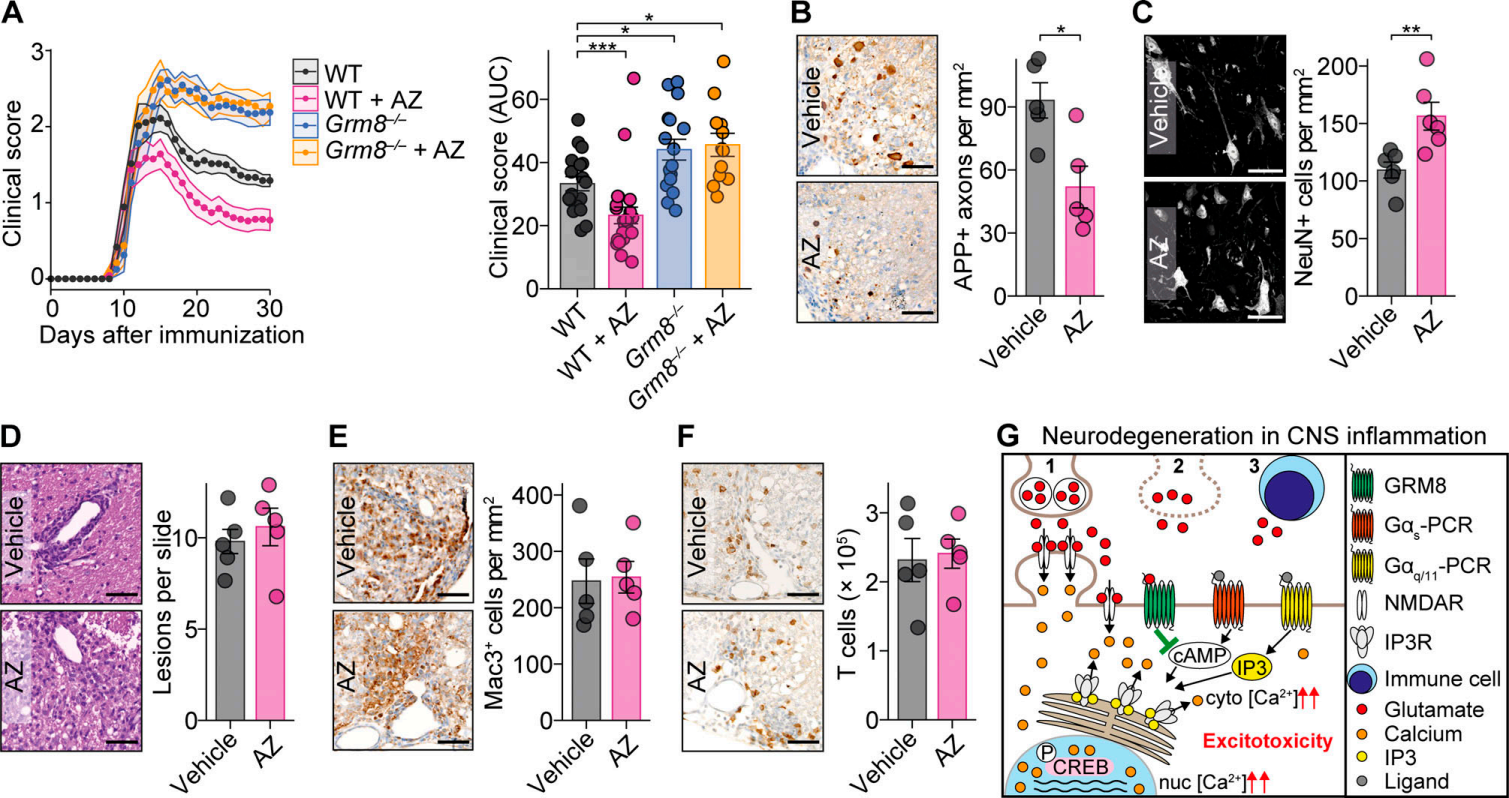
**Activation of Grm8 is neuroprotective in EAE. (A)** Disease course of WT and *Grm8^−/−^* mice that were subjected to EAE and were treated from disease onset with either vehicle or AZ. Pooled data from two independent experiments are shown. WT, *n* = 18; *Grm8^−/−^*, *n* = 16; WT + AZ, *n* = 23; *Grm8^−/−^* + AZ, *n* = 12. **(B and C)** Histopathological quantification of damaged APP-positive axons during acute inflammation 15 d after immunization (B; all groups, *n* = 5) and neuronal loss in chronic phase 30 d after immunization (C; all groups, *n* = 6) of EAE mice that were either vehicle or AZ treated. **(D–F)** Histopathological quantification of inflammatory lesions (D) and Mac3-positive cells (E) and FACS quantification of T cell infiltration (F) during acute phase of EAE 15 d after immunization. All groups, *n* = 5. **(G)** Graphical summary showing detrimental effects of glutamate excess derived by spillover (1), necrotic cell death (2), and secretion from immune cells (3) in CNS inflammation and the counteracting neuroprotective signaling by GRM8 activation. Scale bars, 100 µm. Data are shown as mean values ± SEM. FDR-adjusted Mann–Whitney *U* test was used with *, P < 0.05; **, P < 0.01; ***, P < 0.001.

To then test whether specific activation of GRM8 is neuroprotective in the preclinical mouse model of MS, we subjected WT and *Grm8^−/−^* EAE to daily injections of 1 mg/kg body weight AZ i.p. starting on the day of disease onset. AZ treatment ameliorated the disease course in WT (Fig. S5, A and B) but not in *Grm8^−/−^* mice (pooled data from two independent experiments are shown in Fig. 6 A; results from individual EAE experiments are provided in Fig. S5, C and D, and Table S5), confirming the specificity of the compound in this in vivo model. AZ treatment in WT-EAE was accompanied by fewer APP^+^ damaged axons (Fig. 6 B) and less neuronal loss (Fig. 6 C). Moreover, day of disease onset (Fig. S5 E), proliferation of MOG_35–55_-specific T cells (Fig. S5 F), activation and composition of dendritic cell subsets and T cells (Fig. S5, G and H), the number of lesions, and CNS immune cell infiltration during EAE were not affected by treatment with AZ (Fig. 6, D–F; and Fig. S5 I). Taken together, we conclude that GRM8 activity determines neuronal resilience to inflammation-induced glutamate excitation in this mouse model of MS (Fig. 6 G).

### Glutamate excitotoxicity in MS

**Figure 7.**
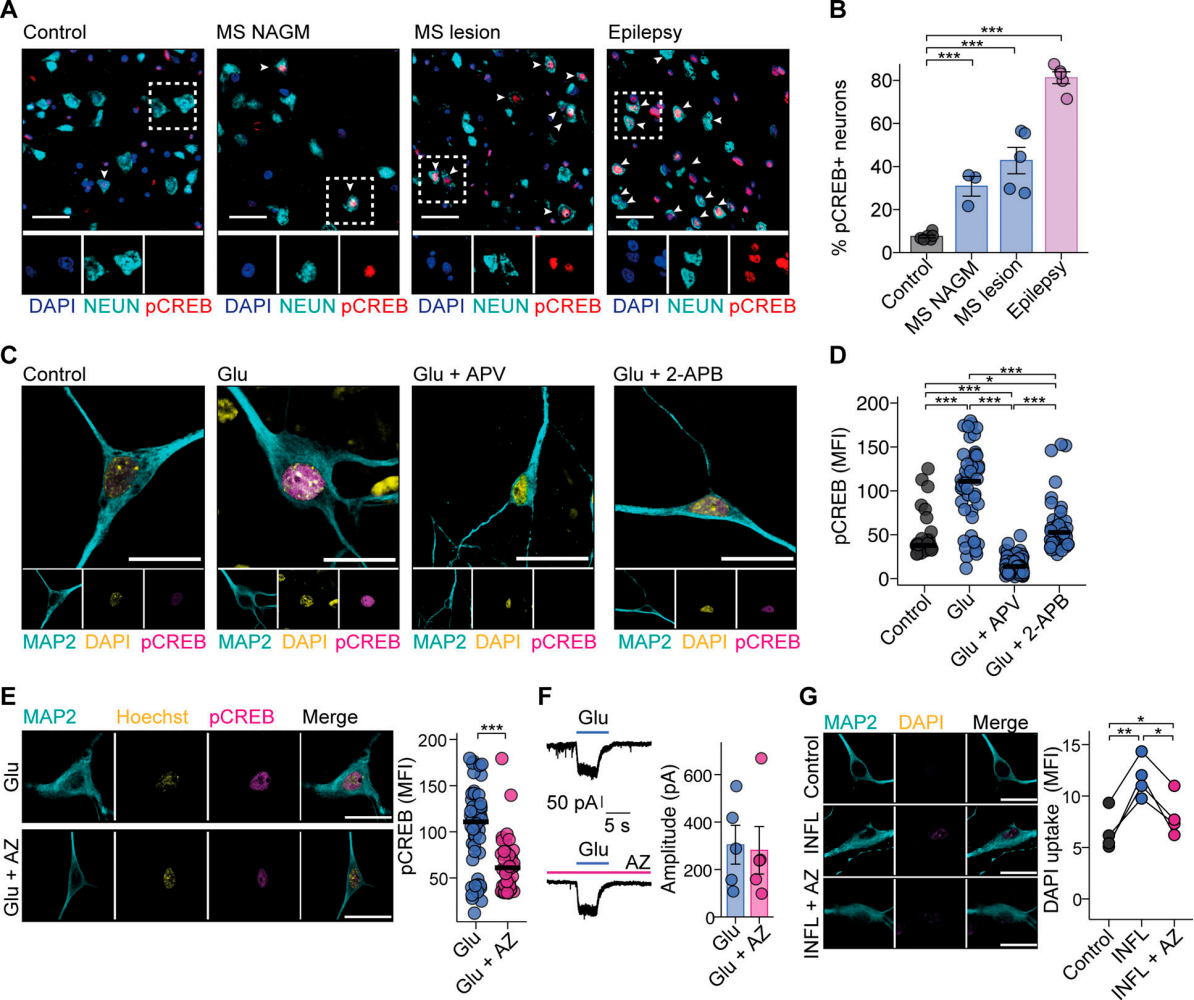
**Activation of GRM8 protects human neurons from glutamate excitotoxicity. (A and B)** Representative images (A) and quantification (B) of percentage of pCREB-positive neurons in brain sections of MS NAGM or cortical MS lesions and epilepsy patients compared with control individuals without neurological diseases. Controls, *n* = 6; MS NAGM, *n* = 3; MS lesions, *n* = 5; epilepsy, *n* = 5. Scale bars, 25 µm. Data are shown as mean ± SEM. FDR-adjusted unpaired two-tailed *t* test was used. **(C and D)** Representative images (C) and quantification (D) of pCREB immunofluorescence in hiPSC neurons that were untreated (control) or that were treated with sham solution, 50 µM APV, or 50 µM 2-APB and subsequently stimulated with 20 µM glutamate (Glu) for 20 min. Control, *n* = 38; glutamate, *n* = 50; Glu + APV, *n* = 78; Glu + 2-APB, *n* = 49. Scale bars, 20 µm. **(E)** pCREB immunofluorescence of hiPSC neurons that were treated with AZ and subsequently exposed to 20 µM glutamate for 20 min. Glu, *n* = 50; Glu + AZ, *n* = 37. Data are shown as median values. Scale bars, 20 µm. **(F)** Patch-clamp recording of inward currents in hiPSC neurons that were incubated in ACSF containing 0.5 µM TTX and 20 µM bicuculline and sham or 1 µM AZ and subsequently were exposed to 10 µM glutamate (Glu) for 4 s. Peak amplitude was used for quantification. Glu, *n* = 5; Glu + AZ, *n* = 5. **(G)** DAPI uptake by hiPSC neurons that were treated with AZ or vehicle and subsequently exposed to IFN-γ, TNF-α, and glutamate. INFL, inflammation. All groups, *n* = 4. Scale bars, 20 µm. FDR-adjusted paired two-tailed *t* test was used. If not stated otherwise, data are shown as mean ± SEM. FDR-adjusted unpaired two-tailed *t* test was used with *, P < 0.05; **, P < 0.01; ***, P < 0.001.

Finally, we investigated whether our mouse findings could be translated back to humans. Therefore, we first assessed *GRM8* expression by RNAscope in situ hybridization and found it similarly expressed in control brain tissue as compared with normal-appearing gray matter (NAGM) and cortical lesions of MS patients (Fig. S5 J). To find molecular evidence of sustained glutamate exposure for neurons in MS, we analyzed the neuronal hallmark of glutamate excitotoxicity: the phosphorylation of serine 133 of cAMP response element-binding protein (pCREB; Hardingham and Bading, 2002). We observed a twofold increase of pCREB-positive neurons in NAGM and a fourfold increase in cortical MS lesions compared with brain sections of non–neurological disease control individuals (Table S6). The strongest pCREB up-regulation was evident in neurons of epilepsy patients (Fig. 7, A and B), representing a pathology that can be attributed to glutamate hyperexcitation (Park et al., 2003; Zhu et al., 2012; Beaumont et al., 2012). Reassuringly, we found that hiPSC-derived excitatory neurons (Fig. S5, K–M; Harberts et al., 2020) strongly induced pCREB after glutamate challenge that was blocked by inhibiting NMDAR or IP3R-dependent calcium release from the ER (Fig. 7, C and D; and Fig. S5 N). This corroborated the importance of calcium release from internal stores also for human glutamate excitotoxicity. Since we found robust *GRM8* expression in hiPSC neurons (Fig. S5 O), we investigated whether GRM8 activation could counteract the pCREB up-regulation that we observed in neurons of MS brains and under excitotoxic treatment. We found that pretreatment with AZ significantly reduced pCREB up-regulation after glutamate application (Fig. 7 E), while AZ alone did not change pCREB baseline levels (Fig. S5 P). Notably, AZ did not affect inward currents of iGluRs (Fig. 7 F), thereby supporting our notion that GRM8 activity induces neuronal resilience by decreasing IP3R sensitivity independently of ion flux through the cell membrane. Last, to more closely mimic MS pathophysiology, we challenged hiPSC neurons with IFN-γ and TNF-α, two abundant cytokines in neuroinflammation (Becher et al., 2017), in combination with glutamate. Also, in response to this challenge, AZ-treated hiPSC neurons showed reduced cell death compared with vehicle-treated hiPSC neurons (Fig. 7 G and Fig. S5 Q). This supports that human GRM8 activation exhibits a neuroprotective effect in an excitatory and inflammatory environment.

## Discussion

Here, we investigated the pathophysiology of neurodegeneration in CNS inflammation and identified glutamate excitotoxicity as a critical component. Excessive activation of NMDAR by elevated glutamate has been proposed to drive several primary neurodegenerative diseases, such as Alzheimer’s disease (Zott et al., 2019) and Parkinson’s disease (Kalia and Lang, 2015), but also MS (Baranzini et al., 2010). In CNS inflammation, glutamate can derive from multiple sources, such as activated Th17 cells that secrete higher levels of glutamate in MS patients’ CSF compared with healthy individuals (Birkner et al., 2020). Furthermore, glutamate is actively released from presynaptic vesicles by neurons in a hypoxic environment and passively set free from dying neurons (Wroge et al., 2012) that can further promote neuronal loss. Increased levels of glutamate in the CNS result in excessive activation of NMDAR and subsequent sustained calcium influx from the extracellular space (Hardingham and Bading, 2010). Thus, ionic disbalance in neurons could drive mitochondrial injury, accumulation of oxidized free radicals, and activation of neuronal regulated cell death (Friese et al., 2014). Although elevated glutamate levels have been described in brains of MS patients by magnetic resonance spectroscopy (Baranzini et al., 2010), it has been unclear whether the sustained increase of glutamate contributes to inflammation-induced neurodegeneration or is a by-product of inflammatory activity (Macrez et al., 2016). Here we used pCREB as a durable molecular marker for continuous glutamate exposure in human neurons and found it strongly up-regulated in glutamate-exposed hiPSC neurons and in MS lesions. This indicates that glutamate excitotoxicity directly contributes to neuronal loss in CNS inflammation. Notably, pCREB levels were also elevated in NAGM, suggesting glutamate-driven neurodegeneration independently of inflammatory lesions in MS. This might contribute to cognitive deficits and cerebral atrophy in MS patients that cannot solely be explained by the spatial distribution of lesions (Kaufmann et al., 2019).

To identify receptors that modulate neuronal resilience during glutamate excitotoxicity in CNS inflammation, we analyzed neuronal receptor networks in single-cell RNA-sequencing data of MS patients. Network construction and subsequent transcriptional network deconvolution (Lachmann et al., 2016) have been successfully used to identify master regulators of numerous cancer types (Alvarez et al., 2016) and neuronal loss in a mouse model of Parkinson’s disease (Brichta et al., 2015). In contrast to available neuronal regulatory networks that used expression of different brain regions from healthy mice (Brichta et al., 2015), we used expression data of healthy and challenged neurons from in vitro primary cultures and in vivo mouse models of neurodegenerative and neuroinflammatory diseases. Our receptor interactome includes stress and steady-state responses as recent advances in transcriptomic analyses revealed an induction of pathways that were traditionally assigned to immune cells, such as IFN signaling, also to be active in neurons during inflammation (Di Liberto et al., 2018; Schattling et al., 2019) and aging (Dulken et al., 2019). We found that, in MS patients, mainly EN-PYRs were affected by a dysregulated receptor interactome that was driven by glutamate activity. This may indicate a neuronal subtype–specific vulnerability to glutamate exposure, as suggested by previous neuropathological studies (Magliozzi et al., 2010; Jürgens et al., 2016).

To counteract the disbalanced receptor interactome, we focused on GRM8 as one of the inhibitory mGluRs. We chose GRM8 as it was associated with MS disease severity (Baranzini et al., 2009; Briggs et al., 2011), and we detected its regulatory network that was associated with neuroaxonal repair to be significantly elevated in pyramidal neurons of MS patients. Thus far, pharmacological inhibition of stimulatory GRM1 and GRM5 signaling did not affect the disease course in EAE (Sulkowski et al., 2013) or the group 2 mGluRs GRM2 and GRM3 (Sun et al., 2013). Moreover, group 3 mGluRs GRM4 and GRM7 are highly expressed in nonneuronal cells, while GRM6 is only expressed in retinal ON-bipolar cells (Peachey et al., 2017), therefore constituting them as unsuitable neuroprotective targets. Similarly, *Grm4* deficiency primarily affects dendritic cells that skew T cell differentiation toward Th17 cells and increases inflammatory activity in EAE (Fallarino et al., 2010). By contrast, GRM8 is an appealing drug target, as it is predominantly expressed in neurons and its activation has been reported to protect undifferentiated neuroblastoma cells against doxorubicin (Jantas et al., 2016) and the mitochondrial toxin MPP4^+^ (Jantas et al., 2014). This suggests that GRM8 activation might exert neuroprotective properties. Other than MS, gene variants of GRM8 have been mostly associated with psychiatric disorders, such as major depressive disorder (Howard et al., 2019) and schizophrenia (Bolonna et al., 2001). In accordance, behavioral studies of mice that are deficient in *Grm8* showed higher levels of anxiety (Duvoisin et al., 2005).

Our data demonstrate that CNS inflammation continuously activates GRM8, as its dependent regulatory network is particularly active in pyramidal neurons of MS patients. This could be interpreted as a neuroprotective countermeasure during chronic glutamate exposure. Accordingly, *Grm8*-deficient neurons were more prone to glutamate excitotoxicity, while pharmacological activation of GRM8 by using AZ was able to further augment neuroprotection in mouse and human neurons. Moreover, daily AZ treatment of mice undergoing EAE profoundly counteracted neurodegeneration. We chose allosteric modulation of GRM8 by AZ, as it provides mechanistic advantages compared with orthosteric agonists. Instead of directly activating GRM8, it increases the physiological signaling initiated from binding of glutamate, with potentially minimized unphysiological receptor activity and the risk for adverse effects (Wootten et al., 2013). Moreover, its structural similarity to other allosteric modulators against Grm1 (Yohn et al., 2020), Grm5 (Haas et al., 2017), and Grm7 (Klar et al., 2015) supports its direct action on the CNS after i.p. treatment. Nevertheless, when considering GRM8 as a therapeutic target, it is important to determine potential unwanted adverse effects. *Grm8* deficiency in mice resulted in mild insulin resistance and weight gain (Duvoisin et al., 2005). Moreover, GRM8 is expressed in glutamatergic neurons of the enteric nervous system and enhances intestinal motility (Tong and Kirchgessner, 2003). Additionally, in the immune system, we found *Grm8* expression exclusively in plasmacytoid dendritic cells in mice. However, we did not observe any differences in immune cell infiltration and the extent of inflammatory lesions during EAE. Further, daily AZ treatment did not affect *Grm8^−/−^* mice, indicating that AZ treatment counteracted inflammation-induced neurodegeneration by specifically promoting *Grm8* activity in neurons.

Mechanistically, GRM8 has been associated with supporting a negative feedback of presynaptic neurotransmitter release. Electrophysiologic recordings of prepulse inhibition (Gosnell et al., 2011) and immunolabeling in the olfactory bulb (Kinoshita et al., 1996) and lateral perforant pathway (Shigemoto et al., 1997) supported this notion of a presynaptic localization. However, its precise subcellular localization was unknown. By expressing fluorescently tagged *Grm8* in cortical neurons, we could now observe pre- and post-synaptic as well as surface localization at neuronal somata. Although overexpression experiments have to be interpreted with caution, the close proximity of GRM8 to excitatory synapses might allow it to monitor and counteract glutamate spillover (Arnth-Jensen et al., 2002) and subsequent hyperexcitation. Despite electrophysiological recordings showing that activation of GRM8 reduced synaptic transmission in the stria terminalis (Gosnell et al., 2011), its mode of action and potential neuroprotective properties have not been investigated.

While glutamate toxicity has been mostly attributed to the influx of neuronal calcium from external sources (Hardingham and Bading, 2002), the contribution of internal calcium stores to excitotoxic cytosolic and nuclear calcium accumulation remains unclear. The ER is the main internal calcium source, and it extends throughout the entire neuron and releases calcium into the cytosol or quenches it to buffer high cytosolic levels (Wu et al., 2017). Calcium release from the ER is mediated by activation of IP3R and ryanodine receptors. Missense mutations of *Itpr1* have been found in patients suffering from spinocerebellar ataxia (Barresi et al., 2017; Hara et al., 2008), and cerebellum-specific deletion of *Itpr1* in mice induces severe ataxia and synaptic loss (Egorova et al., 2016; Kasumu et al., 2012), indicating the importance of ER calcium release for neuronal health. Moreover, β-amyloid aggregate-induced neurotoxicity could also be rescued by blocking IP3R activity (Demuro and Parker, 2013), indicating that unregulated ER calcium release plays an important role in neurodegeneration. Here, we show that IP3R-mediated calcium release from the ER heavily contributes to glutamate-induced excitotoxic calcium accumulation, endorsing its inhibition as an attractive neuroprotective strategy.

We found that activation of GRM8 counteracted this glutamate-induced excitotoxic calcium accumulation by limiting IP3R-dependent calcium release from the ER. Intriguingly, we observed that acute cAMP increase by forskolin strongly enhanced IP3R sensitivity, reinforcing glutamate excitotoxicity. Activation of GRM8 limited cAMP production and thereby decreased IP3R-dependent calcium release from the ER. There are at least two ways that cAMP can regulate IP3R sensitivity: (1) cAMP binding enables PKA to sensitize IP3R1 and IP3R2 or to desensitize IP3R3 by phosphorylation (Vanderheyden et al., 2009) or (2) phosphorylation-independent modulation by direct binding to low-affinity cAMP binding sites of IP3R (Tovey et al., 2008). As we observed an immediate calcium release from the ER by simultaneously applying forskolin and glutamate, and as inhibition of PKA did not reduce cAMP-enhanced calcium release, we assume that this supports a direct effect that is independent of phosphorylation (Gelens and Saurin, 2018). Thus, pathological cAMP accumulation by dysregulated metabotropic signaling likely contributes to neurodegeneration by promoting excessive calcium release from the ER through direct sensitization of IP3R. Although HEK cells (Konieczny et al., 2017) and osteoblasts (Buckley et al., 2001) react differently to IP3 than to IP3 together with cAMP, the direct interaction site of cAMP with different IP3R isotypes is currently unknown. However, this suggests that cAMP-mediated IP3R sensitization may be a widespread mechanism in different cell types that could be modulated by G protein–coupled receptor–targeted drugs (Hauser et al., 2017). Our data infer that IP3R sensitivity is a crucial determinant of neuronal calcium homeostasis and integrity, which are directly modulated by the druggable GRM8. Thus, the interplay between metabotropic signaling and internal calcium stores emerges as a central pathophysiological mechanism warranting further characterization in other neurodegenerative processes.

In summary, we demonstrate that GRM8 is a decisive player in an endogenous feedback mechanism to limit glutamate-induced excitotoxic calcium accumulation in neurons. Our findings are a rare example of a neuroprotective pathway sensu stricto that increases neuronal resilience without impacting the immune response during CNS inflammation (Friese et al., 2014). This commends GRM8 activation as a valuable therapeutic approach to counteract inflammation-driven neurodegeneration in MS and other neurological diseases that involve glutamate excitotoxicity.

## Materials and methods

### Mice

All mice (C57BL/6J WT [The Jackson Laboratory]; C57BL/6J *mGluR8^−/−^* [Duvoisin et al., 2005]; and FVB/NRJ Epac1-PLN [Sprenger et al., 2015]) were kept under specific pathogen–free conditions in the central animal facility of the University Medical Center Hamburg-Eppendorf (UKE). We used adult mice (6–20 wk old) of both sexes; mice were sex and age matched in all experiments. We did not observe sex-specific differences in any of the experiments; therefore, the sexes were reported together.

### EAE

We immunized mice subcutaneously with 200 µg MOG_35–55_ peptide (Schafer-N) in CFA (Difco; catalog no. DF0639-60-6) containing 4 mg ml^−1^ *Mycobacterium tuberculosis* (Difco; catalog no. DF3114-33-8). In addition, we injected 200 ng pertussis toxin (Calbiochem; catalog no. CAS70323-44-3) i.p. on the day of immunization and 48 h later. We scored animals daily for clinical signs by the following system: 0, no clinical deficits; 1, tail weakness; 2, hindlimb paresis; 3, partial hindlimb paralysis; 3.5, full hindlimb paralysis; 4, full hindlimb paralysis and forelimb paresis; 5, premorbid or dead. Animals reaching a clinical score ≥4 were euthanized according to the regulations of the local Animal Welfare Act. Where indicated, animals were injected i.p. with 1 mg kg^–1^ body weight AZ 12216052 (Tocris; catalog no. 4832) starting from the day of disease onset. We used littermate controls in all EAE experiments. AZ was prediluted in DMSO, and the final injection consisted of 10% DMSO ± AZ, 40% polyethylene glycol (Thermo Fisher Scientific; catalog no. P/3676/08), and 50% Dulbecco’s PBS (Pan Biotech). The results and number of animals from independent EAE experiments are provided in Table S5. For recall assays (described below), mice were treated with DMSO vehicle control or AZ from day 3 after immunization for 6 d and were used for experiments 9 d after immunization. The investigators were blind to the genotype and treatment in the EAE experiments.

### hiPSC-derived neurons

We maintained hiPSCs (ZIPi013-B; Tandon et al., 2018) under feeder-free conditions on Matrigel (Corning)-coated plates in mTeSR1 medium (STEMCELL Technologies; catalog no. 85850). For neuronal induction, we dissociated hiPSCs with Accutase and seeded them at a density of 3 × 10^6^ cells per well on AggreWell800 plates (10,000 cells per embryoid body; STEMCELL Technologies) in SMADi neural induction medium (STEMCELL Technologies; catalog no. 08582) supplemented with 10 µM Y-27632 (STEMCELL Technologies; catalog no. 72302). On day 6, embryoid bodies were harvested and cultivated on Matrigel-coated plates in SMADi neural induction medium for 12 d. Newly formed neural rosettes were manually picked and cultured for another 4 d. To release neural precursor cells (NPCs), neural rosettes were dissociated with Accutase and were maintained for several passages at high density in Neural Progenitor Medium (STEMCELL Technologies; catalog no. 05833) on Matrigel-coated plates. We differentiated hiPSC-derived NPCs into neurons as previously described (Brennand et al., 2011; Djuric et al., 2015; Zhang et al., 2016) with some modifications. Briefly, NPCs were seeded at a density of 5 × 10^4^ cells/cm^2^ in Neural Progenitor Medium onto poly-L-ornithine/laminin–coated plates. After 24 h, we replaced the medium by neural differentiation medium (day 0 of differentiation) composed of Neurobasal Plus Medium (Gibco BRL; catalog no. A3582901) containing 1× B27 Plus Supplement (Gibco BRL; catalog no. A3582801), 1× N2 Supplement-A (STEMCELL Technologies; catalog no. 07152), 1× MEM nonessential amino acids (Gibco BRL; catalog no. 11140050), 1 µg ml^–1^ laminin (Sigma-Aldrich; catalog no. 11243217001), 1 µM dibutyryl-cAMP (STEMCELL Technologies; catalog no. 73882), 10 ng ml^–1^ L-ascorbic acid (STEMCELL Technologies; catalog no. 72132), 10 ng ml^–1^ brain-derived neurotrophic factor (STEMCELL Technologies; catalog no. 78005), and 10 ng ml^–1^ glia-derived neurotrophic factor (STEMCELL Technologies; catalog no. 78058). To promote a glutamatergic neuronal cell type, 5 µM cyclopamine (STEMCELL Technologies; catalog no. 72072) was additionally added to the medium during the first week of differentiation. In the second week, we supplemented neuronal differentiation medium with 2 µM cytarabine (Sigma-Aldrich; catalog no. BP383) in order to reduce proliferation of nonneuronal cells. On day 14, the cells were detached using Accutase and reseeded onto 12-mm-diameter coverslips. Thereafter, cells were maintained for up to 18–20 wk to increase maturity.

### Primary mouse neuronal cultures

For primary cortical cultures, we euthanized pregnant C57BL/6J, FVB/NRJ Epac1-PLN, or *mGluR8^+/−^*mice. To ensure comparability between genotypes, we used only embryos from heterozygous breeding. We reserved tissue of each embryo for genotyping and isolated the cortex, dissociated, and plated cells at a density of 10^5^ cells per 1 cm^2^ on poly-D-lysine–coated wells (5 µM; catalog no. A-003-M; Sigma-Aldrich). If not stated otherwise, cells were maintained in Neurobasal Plus Medium (supplemented with B27 Plus, penicillin, streptomycin, and L-glutamine; Gibco BRL; catalog no. A3582901) at 37°C, 5% CO_2_, and a relative humidity of 98% and treated with 1 µM cytarabine (Sigma-Aldrich; catalog no. BP383) at 1 d in vitro (1 div) to inhibit glial cell proliferation. If no cytarabine was applied, cells were maintained in neurobasal medium (supplemented with B27, penicillin, streptomycin, and L-glutamine; Gibco BRL). Throughout, we used cultures after 14–23 div for experiments.

### GSEA

We downloaded published expression data from the Gene Expression Omnibus (GEO) and derived murine neuronal stress signatures from GSE10470, GSE22087, GSE22465, GSE22997, GSE109177, and GSE122121. We selected glutamate-regulated genes from Zhang et al. (2007); a murine dataset of noninflamed and inflamed neurons from EAE derived from GSE104897; and human datasets of healthy individuals and MS patients from GSE10800, GSE26927, GSE118257, and PRJNA544731. We analyzed microarray datasets by the standard *limma* pipeline (Ritchie et al., 2015). We contrasted stressed neurons against control neurons or MS brain tissue against nondiseased control brain tissue, respectively. We analyzed RNA-sequencing datasets by a standard *DESeq2* pipeline (Love et al., 2014). We identified neuronal transcript counts from single-nucleus sequencing datasets by the annotation provided by GEO and summed up counts for each gene and for each individual. The resulting expression matrix consisted of the neuronal transcription profile of every individual. We analyzed differential gene expression (DE) between MS patients and nondiseased individuals by *DESeq2*. To create ranked gene lists, we arranged DE results from healthy MS comparisons by *limma*-derived moderated *t*-statistics for GSE10800 and GSE26927 or by *DESeq2*-derived Wald statistics for GSE104897, GSE118257, and PRJNA544731. For neuronal stress signatures, we only considered genes with a positive fold change and false discovery rate (FDR)–adjusted P < 0.05. When >100 genes fulfilled the criteria, only the top 100 most significant genes were used to get comparable gene set sizes. When genes were represented by multiple probes, the one with the highest absolute deviation around the median was considered. To avoid batch effects and interspecies differences of gene expression, we did not directly compare differentially regulated genes but rather assessed differentially regulated biological themes that consist of gene groups that represent biological functions across species. Therefore, we performed GSEA using clusterProfiler (Yu et al., 2012).

### Regulatory network analysis

Raw read counts of 502 neuron-specific mRNA sequencing datasets of in vitro healthy and challenged neuronal cultures and in vivo mouse models of psychiatric, neurodegenerative, neuroinflammatory, and metabolic diseases were retrieved from the Sequence Read Archive and were aligned to the mouse reference genome (mm10) using STAR version 2.4 (Dobin et al., 2013) with default parameters; overlap with annotated gene loci was counted with featureCounts version 1.5.1 (Liao et al., 2014). The regulatory network was reverse engineered using ARACNe (Lachmann et al., 2016). ARACNe was run with 100 bootstrap iterations using all probes that mapped to a set of 1,101 mouse transmembrane receptors, which were defined as genes as members of Gene Ontology identifier GO:0003700, “transmembrane signaling receptor activity,” and its respective offspring. Olfactory receptor genes were excluded from the analysis. As recommended for bootstrap ARACNe analysis, we used 0 data processing inequality tolerance and a threshold for mutual inference P < 10^−7^. Genome-wide expression signatures of neuron subtype–specific changes in MS patients compared with nondiseased controls were computed as described above from Schirmer et al. (2019). We used the annotation provided by GEO for filtering different neuron subtypes. DE between MS patients and nondiseased controls was tested by *DESeq2* (Love et al., 2014). The receptor interactomes of different neuron subtypes from MS patients compared with nondiseased controls were computed by Virtual Inference of Protein-activity by Enriched Regulon (Viper; Alvarez et al., 2016) using ranked gene lists of each neuronal subtype from MS patients compared with nondiseased controls and the regulatory transmembrane receptor network we created as input. The FDR-adjusted P value and normalized enrichment score (NES) were computed by comparison with a null model that was generated by permuting the samples uniformly at random 1,000 times. Subsequent enrichment analysis was performed using clusterProfiler (Yu et al., 2012).

### Chemicals

The used chemicals and the respective function, supplier, catalog number, and concentration that we used in vitro are depicted in Fig. S3 I and Table S7.

### RNAscope in situ hybridization

We performed RNAscope fluorescent in situ hybridization using the RNAscope Fluorescent Multiplex Kit V2 (Advanced Cell Diagnostics; catalog no. 323100) according to the manufacturer’s protocol. Probes against human Hs-*Snap25*-C3 (catalog no. 518851-C3) and Hs-*Grm8* (catalog no. 563351) were commercially available from Advanced Cell Diagnostics, Inc. RNAscope human samples were scanned using the Pannoramic 250 FLASH II (3DHISTECH) Digital Slide Scanner at 20× magnification. *GRM8^+^SNAP25^+^* neurons were quantified by a blinded experimenter using Pannoramic Viewer software (3DHISTECH) and Fiji (National Institutes of Health [NIH] image analysis software) or with a custom-made script, which was based on Cognition Network Language (Definiens Cognition Network Technology; Definiens Developer XD software).

### Immunohistochemistry, immunohistopathology, and immunocytochemistry

The used primary and secondary antibodies and the respective antigen, host species, supplier, catalog number, and dilution are listed in Table S8. Human CNS tissue was fixed with 4% paraformaldehyde and embedded in paraffin as described previously (Kreutzfeldt et al., 2013). To prevent unspecific binding, we performed antigen retrieval. Human sections were scanned using the Pannoramic 250 FLASH II (3DHISTECH) Digital Slide Scanner at 20× magnification. Positive signals in a field of view of 1.2 mm^2^ were quantified using CaseViewer software (3DHISTECH). Mouse spinal cord tissue was obtained and processed as described previously (Schattling et al., 2012). Mouse sections were analyzed with a Zeiss LSM 700 confocal microscope. For histopathology, we used hematoxylin (blue color) and immunolabeling that we visualized using the avidin–biotin complex technique with 3,3′-diaminobenzidine (brown stain). We analyzed slides with a NanoZoomer 2.0-RS digital slide scanner and NDP.view2 software (Hamamatsu). We quantified CD3- and Mac3-positive cells as well as APP deposits in the white matter tract of the spinal cord using a customized counting mask with Fiji (ImageJ). NeuN-positive cells (neurons) were manually counted in the ventral horn outflow tract of the spinal cord. For Luxol fast blue staining, we quantified the positive area in the white matter of the spinal cord using a customized counting mask with Fiji (ImageJ). Analysis conditions were standardized across all conditions. At least three images were analyzed per animal, and the mean per animal was used for subsequent statistical comparisons. For immunocytochemistry of hiPSC neurons and mouse neurons, we cultivated cultures on 12-mm-diameter coverslips, fixed them with 4% paraformaldehyde, incubated them in 10% normal donkey serum containing 0.1% Triton X-100, and subsequently performed immunolabeling. For surface staining, we incubated transfected cultures in ice-cold medium for 30 min with the primary anti-GFP antibody (1:200), subsequently fixed them, and applied the secondary antibody before permeabilization (1:500) in 10% NDS. Afterward, the staining protocol was continued as described above. To visualize neuronal morphology, we used actin-stain 555 phalloidin (1:100; Cytoskeleton; catalog no. PHDH1-A) and actin-stain 670 phalloidin (1:100; Cytoskeleton; catalog no. PHDN1-A). To measure the influence of glutamate on human neuronal pCREB regulation, we pretreated hiPSC neuron cultures with 1 µM AZ, 50 µM 2-amino-5-phosphonovaleric acid (APV), 50 µM 2-APB, or 0.1% DMSO (vehicle) and stimulated them for 20 min with 20 µM glutamate or 0.1% PBS. We visualized stained cells by confocal microscopy (see above).

### RealTime-Glo cell viability assay

We mixed RealTime-Glo (Promega; catalog no. G9711) MT cell viability substrate and NanoLuc Enzyme together, added it to neuronal cultures, and incubated them for 5 h for equilibration of luminescence signal before the respective treatments were applied. We recorded luminescence with a Spark 10M multimode microplate reader (Tecan) at 37°C and 5% CO_2_ every 30 min over a total time period of 20–24 h. We used at least five technical replicates per condition. For analysis, every well’s data point was normalized to its last value before the stressor was added and then normalized to the mean of the control wells for every time point. Thereby we controlled for well-to-well seeding variability. For statistical analysis, we compared either area under the curve (AUC) or endpoint.

### CellTiter-Glo cell viability assay

24 h after stimulation of neuronal cultures, the CellTiter-Glo Luminescent Cell Viability Assay (Promega; catalog no. G7570) was performed according to the manufacturer’s protocol. We recorded luminescence with a Spark 10M multimode microplate reader (Tecan).

### Real-time PCR

We reverse transcribed RNA to cDNA with the RevertAid H Minus First Strand cDNA Synthesis Kit (Thermo Fisher Scientific) according to the manufacturer’s instructions. We analyzed gene expression by real-time PCR performed in an ABI Prism 7900 HT Fast Real-Time PCR System (Applied Biosystems) using TaqMan Gene Expression Assays (Thermo Fisher Scientific) for *Grm8* (Mm00433840_m1), *Grm4* (Mm01306128_m1), *Grm6* (Mm00841148_m1), *Grm7* (Mm01189424_m1), *Fos* (Mm00487425_m1), *Bdnf* (Mm00432069_m1), *Grin1* (Mm00433790_m1), *Grin2a* (Mm00433802_m1), *Grin2b* (Mm00433820_m1), *Gria1* (Mm00433753_m1), *Grik1* (Mm00446882_m1), *Slc1a2* (Mm00441457_m1), *Itpr1* (Mm00444937_m1), *Itpr2* (Mm00439907_m1), *Itpr3* (Mm01306070_m1), *Tbp* (Mm00446971_m1), *GRM8* (Hs00945353_m1), and *TBP* (Hs00427620_m1). We calculated gene expression as 2^–ΔCt^ relative to *Tbp* (mouse) or *TBP* (human) as the endogenous control.

### Isolation of CNS-infiltrating immune cells and flow cytometry

CNS-infiltrating immune cells from EAE animals during the inflammatory phase 12–17 d after immunization were isolated and quantified as we described previously (Ufer et al., 2016). We stained single-cell suspensions in the presence of TruStain Fc receptor block (BioLegend) and used Alexa Fluor 750 NHS Ester (Invitrogen) for live/dead discrimination. The antibodies and the respective antigen, host species, supplier, catalog number, clone, and dilution are listed in Table S8. Data were acquired on an LSR II FACS analyzer (BD Biosciences).

### Recall assay

For antigen-specific recall assays, 9 d after immunization of the mice, 2.5 × 10^5^ draining inguinal lymph node cells were prepared and cultured in 96-well round-bottom plates for 72 h with the indicated concentrations of MOG_35–55_ peptide, a vehicle control, or plate-coated anti-CD3ε (1 µg/ml; BioLegend; catalog no. 100340) together with soluble anti-CD28 (1 µg/ml; BioLegend; catalog no. 102116) as a positive control. During the last 16 h of culture, cells were pulsed with 1 µg/ml BrdU (catalog no. 423401). Single-cell suspensions were stained for surface antigens in the presence of TruStain Fc receptor block (BioLegend), and Fixable Viability Stain 780 (BD Biosciences; catalog no. 565388) was used to discriminate dead cells. Cells were fixed (fixation buffer; BioLegend; catalog no. 420801) and permeabilized using 0.5% Triton X-100, followed by incubation with 40 KU/ml DNase I (Merck; catalog no. 260913-10MU) in PBS with Ca^2+^ and Mg^2+^ for 1 h at 37°C. After DNA digestion, incorporated BrdU was detected by incubation with an anti-BrdU AF647-coupled antibody. The antibodies and the respective antigen, host species, supplier, catalog number, clone, and dilution are listed in Table S8. Data were acquired on an LSR II FACS analyzer (BD Biosciences). Representative gating strategies will be provided upon request.

### DAPI cell toxicity assay

hiPSC neurons were incubated with either 1 µM AZ or 0.1% DMSO for 1 h and then stimulated with 200 µM glutamate, 100 ng ml^–1^ IFN-γ (PeproTech; catalog no. 315-05), and 50 ng ml^–1^ TNF-α (PeproTech; catalog no. 315-01A). After 24 h, we added 5 µM DAPI (Invitrogen) to the culture for 10 min and performed immunostaining as described above for a neuronal marker (Map2; see above) and propidium iodide (PI; 1:1,000; BioLegend; catalog no. 421301) to identify all nuclei. We used neuronal nuclei as the region of interest to quantify DAPI uptake by mean fluorescence intensity (MFI) as a measure of neuronal cell damage (Fig. S5 Q).

### Calcium imaging

We seeded primary neuronal cultures on either the Ibidi 60 µ-Dish Quad (catalog no. 80411) or High (catalog no. 81158) with a glass bottom. To measure cytosolic calcium changes, we infected neuronal cultures with an AAV7 containing pAAV-Syn-GCamp6f-WPRE-SV40 (Chen et al., 2013; Addgene; 100837) at 8–12 div with a 10,000–20,000-fold multiplicity of infection. AAV particles were produced according to the standard procedures of the UKE vector facility. We acquired images with a confocal LSM 700 laser scanning confocal microscope (Zeiss) every 0.48 s with 20× magnification in an imaging chamber maintaining 37°C and 5% CO_2_. Infected cultures were imaged in the respective culture medium. We isolated mGluR signaling by applying 25 µM bicuculline, 2 µM CGP 55845, 50 µM APV, 20 µM 2,3-dioxo-6-nitro-1,2,3,4-tetrahydrobenzo[f]quinoxaline-7-sulfonamide disodium salt (NBQX) and 20 µM DL-threo-b-benzyloxyaspartate (DL-TBOA) and subsequently applied 20 µM glutamate. Response-modifying chemicals were applied simultaneously to the isolation mix. Since Gcamp6f is not expressed in the nucleus (Dana et al., 2019), we used Fluo-4 acetoxymethyl ester (Thermo Fisher Scientific; catalog no. 14201) to measure nuclear calcium. For that, we incubated neuronal cultures in medium with 5 µM Fluo-4 acetoxymethyl ester for 30 min at 37°C and 5% CO_2_. Then, cells were rinsed three times and left to equilibrate in imaging buffer (10 mM glucose, 140 mM NaCl_2_, 1 mM MgCl_2_, 5 mM KCl, 20 mM Hepes, and 2 mM CaCl_2_, pH 7.4) for at least 30 min before imaging. If indicated, 1 µM tetrodotoxin (TTX) was added to electrically silence the cultures. In general, we recorded the first 5–10 min of baseline activity before applying the indicated chemicals. At the end of recording, we applied 10 µM ionomycin to induce maximum cellular calcium response that was used for normalization. Specific assay details and concentrations can be found in the respective figure legends. For data analysis, we measured mean fluorescence values of every cell using Fiji software (NIH) and normalized it to either the maximal calcium response after ionomycin challenge (indicated as F/F_Max_) or to the mean fluorescence of the baseline (indicated as F/F_Baseline_). For each cell, we calculated maximal, minimal, mean, and AUC of the calcium response using a custom R script. If not stated otherwise, AUC was used for statistical comparisons.

### cAMP imaging

We seeded primary neuronal cultures from pregnant FVB/NRJ Epac1-PLN mice on 25-mm-diameter coverslips and imaged them at div 21. The imaging setup has been described in detail elsewhere (Sprenger et al., 2012). Briefly, we washed coverslips twice with imaging buffer (see above) and subjected them to mGluR isolation (see above) with additional treatment of either 0.1% DMSO (vehicle), 50 µM 2-APB, or 1 µM AZ. After a stable FRET ratio was reached, we recorded for 1 min as a baseline and subsequently stimulated cultures with 10 µM glutamate for at least 10 min. As a viability control, 50 µM forskolin and 50 µM 3-isobutyl-1-methylxanthine were finally added. We recorded FRET measurements using an inverted fluorescent microscope (Nikon Ti) and Fiji software. The FRET donor CFP was excited at 440 nm using a CoolLED light source. The exposure time was 10 ms, and images in CFP and YFP emission were acquired every 5 s. For data analysis, we normalized YFP/CFP ratios to mean fluorescence of the baseline measurement. For each cell, we calculated maximal, minimal, mean, and AUC using a custom R script.

### Neuronal nuclei isolation and flow cytometry

Nuclei of mouse spinal cords were isolated with the Nuclei Isolation Kit (Sigma-Aldrich; catalog no. NUC101) according to the manufacturer’s protocol. To obtain neuronal nuclei, we stained nuclei with PI (1:2,000; see above) and a primary labeled antibody directed against NeuN (1:500). Then we sorted PI^+^NeuN^+^ nuclei by using a BD Aria III cell sorter (BD Biosciences). We processed RNA for real-time PCR as described above.

### Electrophysiological recordings of hiPSC neurons

For patch-clamp experiments, artificial cerebrospinal fluid (ACSF) with low magnesium was used as an extracellular solution. ACSF was oxygenated during experiments with 95% (vol/vol) O_2_ and 5% CO_2_ (pH 7.3–7.4) containing the following (in mM): 125 NaCl, 2.5 KCl, 1.25 NaH_2_PO_4_, 25.0 NaHCO_3_, 0.2 MgCl_2_, 2.0 CaCl_2_, and 25 glucose. The internal recording pipette solution contained (in mM): 120 KMeSO_4_, 20 KCl, 10 Hepes, 0.2 EGTA, 2 MgCl_2_, 4 Na_2_ATP, and 0.3 Na_2_GTP; pH was adjusted to 7.3 with KOH. If indicated in the respective figures, 0.5 µM TTX, 50 µM APV, 20 µM 6-cyano-7-nitroquinoxaline-2,3-dione (CNQX), 20 µM bicuculline, 10 µM glutamate, or 1 µM AZ was added to ACSF or was applied for 4–8 s. All experiments were done at room temperature (22–25°C). Somatic whole-cell voltage-clamp and current-clamp recordings were obtained from visually identified hiPSC neurons with a 40× objective of a Zeiss Axioskop 2 FS Plus microscope. Borosilicate glass capillaries (GC150F-10; Harvard Apparatus) were pulled (Flaming/Brown micropipette puller, model P-97; Sutter Instrument) and had a resistance of 3–5 MΩ when filled with internal solutions. Membrane currents and action potentials were recorded with an EPC9 amplifier (HEKA Elektronik) using Patchmaster software. Only recordings with an access resistance <25 MΩ were evaluated. Series resistance was compensated to 70–80%. Neurons were perfused continuously (1–1.5 ml min^–1^) with carbonated ACSF.

### Vector construction and transfection

To visualize Grm8 localization, we inserted EGFP at the N-terminal extracellular domain next to the 33–amino acid–long signal peptide. Sequentially, EGFP (Primer_f_1, Primer_r_2 from pcDNA3-EGFP), *Grm8* signal peptide (Oligo_f_1, Oligo_f_2), and mm*Grm8* without signal peptide (Primer_f_3, Primer_r_4 from mouse brain cDNA) were inserted into a temporary backbone. Primers, oligonucleotides, and the respective restriction sites are listed in Table S9. For the final construct, we used a modified pAAV-hSyn-EGFP as a backbone. pAAV-hSyn-EGFP was a gift from Bryan Roth (Department of Pharmacology, University of North Carolina at Chapel Hill, Chapel Hill, NC; Addgene 50465; http://n2t.net/addgene:50465; Research Resource Identifier Addgene_50465). First, EGFP was replaced with a multiple cloning site (Oligo_f_3, Oligo_f_4), and then SP-EGFP-*Grm8* (Primer_f_5, Primer_r_4) was inserted, resulting in the pAAV-hSyn-SP-EGFP-mm*Grm8* construct, which we used to transfect primary neuronal cultures alone or together with a tdTomato expression construct at div 1 with 500 ng DNA and Lipofectamine 3000 (Invitrogen; catalog no. L3000001) according to the manufacturer’s protocol.

### Statistical analysis

The statistical analyses applied during the bioinformatics analysis are detailed in the respective sections of the article. Flow cytometric data were analyzed by using FlowJo software (FlowJo LLC). Images were analyzed by using Fiji software (NIH). Patch-clamp data were analyzed by using Fitmaster (HEKA Elektronik) and Igor Pro 6.03 (Wavemetrics). Experimental data were analyzed within the R environment (version 1.2.5001) on a Mac OS X. Unless stated otherwise, the data are presented as mean ± SEM, and differences between two experimental groups were determined by using unpaired, two-tailed Student’s *t* tests and were FDR corrected for multiple comparisons. Statistical analysis of the clinical scores in the EAE experiments was performed by applying a Mann-Whitney *U* test to the AUCs for each animal. The exact number of experiments is provided in the figure legends. Significant results are indicated by P < 0.05, P < 0.01, and P < 0.001.

### Data and materials availability

The datasets analyzed during the study are available in the GEO database, and the corresponding accession numbers are listed in the Material and methods section. Signature gene lists for neuronal stressors are provided in Table S1; Sequence Read Archive identifier and fastq download links of datasets used for ARACNe are listed in Table S2; input receptors for ARACNe are shown in Table S3; and the neuronal receptor network is provided in Table S4. The R code used for live-cell imaging analysis, GSEA, and the transmembrane receptor regulatory network is available from the corresponding author on reasonable request.

### Study approval

All animal care and experimental procedures were performed according to institutional guidelines and conformed to the requirements of the German Animal Welfare Act. Ethical approvals were obtained from the State Authority of Hamburg, Germany (approval no. 15/81, ORG713). As human tissue could no longer be assigned to a human being, the analyses did not constitute a “research project on humans” in the sense of section 9, paragraph 2, of the Hamburg Chamber of Commerce Act for the Health Professions and therefore did not require consultation in accordance with section 15, paragraph 1, of the Professional Code of Conduct for Physicians in Hamburg. The use of hiPSCs was approved by the ethics committee of the Kiel University, Germany (A145/11), and is further described at https://www.sciencellonline.com/technical-support/ethical-statement.html.

### Online supplemental material

Fig. S1 shows the expression of MS-associated glutamate receptors and neuronal receptor interactomes in different neuronal subsets of MS patients and healthy controls. Fig. S2 characterizes *Grm8* mRNA expression in different tissues and cell types and that *Grm8^−/−^* and WT neurons do not differ in baseline viability and glutamate receptor expression. Fig. S3 shows that *Grm8^−/−^* neurons have enhanced calcium accumulation in different glutamate-dependent stress assays and further supports that metabotropic glutamate signaling depends on IP3R signaling and is modulated by cAMP. Fig. S4 shows that *Grm8^−/−^* and WT animals do not differ in baseline axonal and synaptic density and the immune cell infiltration during the acute phase of EAE but have more demyelination. Fig. S5 shows that Grm8 activation by chronic application of AZ does not alter the immune response in the acute phase of EAE and electrophysiological recordings that support the excitatory differentiation of hiPSC neurons and neuron-specific *Grm8* expression in hiPSC neuronal cultures. Table S1 lists neuronal stress signature genes that were used for GSEA in Fig. 1. Table S2 lists datasets, identifiers, and fastq download links for datasets that were used for ARACNe. Table S3 includes Ensembl gene names of receptors that were used as input for ARACNe. Table S4 shows the neuronal receptor network output from ARACNe. Table S5 lists the results and the number of animals used in individual EAE experiments. Table S6 summarizes clinical data for brain specimens. Table S7 lists chemicals, Table S8 lists antibodies, and Table S9 lists primers and oligonucleotides that we used for creating overexpression constructs.

## Supplementary Material

Table S1 lists neuronal stress signature genes.

Table S2 lists datasets and identifiers used for ARACNe.

Table S3 lists input Ensembl gene name lists of receptors for ARACNe.

Table S4 lists neuronal receptor networks.

Table S5 lists the results and the number of animals used in individual EAE experiments.

Table S6 summarizes clinical data for brain specimens.

Table S7 lists chemicals used in this study.

Table S8 lists antibodies used in this study.

Table S9 lists primers and oligonucleotides that we used for creating overexpression constructs.
