# Supplementary material for: Neuronal metabotropic glutamate receptor 8 protects against neurodegeneration in CNS inflammation
Source: J Exp Med. 2021 Mar 4;218(5):e20201290. doi: 10.1084/jem.20201290 (PMC7938362; doi:10.1084/jem.20201290)
Supplement: Table S5 — lists the results and the number of animals used in individual EAE experiments. [file JEM_20201290_TableS5.docx]

Table S5. Results of individual EAE experiments

| Figure | Genotypes | Treatment | Conditions (*n*) | Comparisons | P value (AUC) | P value (last day) |
| --- | --- | --- | --- | --- | --- | --- |
| Fig. S4 A | WT, *Grm8^–/–^* | No | WT (7), *Grm8^–/–^* (10) | WT vs. *Grm8^–/–^* | 0.03 | 0.04 |
| Fig. S4 B | WT, *Grm8^–/–^* | No | WT (9), *Grm8^–/–^* (13) | WT vs. *Grm8^–/–^* | 0.04 | 0.002 |
| Fig. S4 C | WT, *Grm8^–/–^* | No | WT (11), *Grm8^–/–^* (9) | WT vs. *Grm8^–/–^* | 0.12 | 0.19 |
| Fig. S5 A | WT | Vehicle, AZ | WT + vehicle (26), WT + AZ (23) | WT + vehicle vs. WT + AZ | 0.03 | 0.04 |
| Fig. S5 C | WT, *Grm8^–/–^* | Vehicle, AZ | WT + vehicle (10), WT + AZ (12), *Grm8^–/–^* + vehicle (8), *Grm8^–/–^* + AZ (6) | WT + vehicle vs. WT + AZ | 0.005 | 0.002 |
|  |  |  |  | WT + vehicle vs. *Grm8^–/–^* + vehicle | 0.35 | 0.001 |
|  |  |  |  | *Grm8^–/–^* + vehicle vs. *Grm8^–/–^* + AZ | 0.85 | 0.24 |
|  |  |  |  | WT + AZ vs. *Grm8^–/–^* + vehicle | 0.001 | <0.001 |
|  |  |  |  | WT + vehicle vs. *Grm8^–/–^* + AZ | 0.42 | 0.001 |
|  |  |  |  | WT + AZ vs. *Grm8^–/–^* + AZ | 0.001 | <0.001 |
| Fig. S5 D | WT, *Grm8^–/–^* | Vehicle, AZ | WT + vehicle (8), WT + AZ (11), *Grm8^–/–^* + vehicle (8), *Grm8^–/–^* + AZ (6) | WT + vehicle vs. WT + AZ | 0.07 | 0.02 |
|  |  |  |  | WT + vehicle vs. *Grm8^–/–^* + vehicle | 0.02 | 0.002 |
|  |  |  |  | *Grm8^–/–^* + vehicle vs. *Grm8^–/–^* + AZ | 0.75 | 0.9 |
|  |  |  |  | WT + AZ vs. *Grm8^–/–^* + vehicle | 0.009 | 0.002 |
|  |  |  |  | WT + vehicle vs. *Grm8^–/–^* + AZ | 0.004 | 0.004 |
|  |  |  |  | WT + AZ vs. *Grm8^–/–^* + AZ | 0.01 | 0.004 |

P values of AUC and scores from last day of EAE were determined by using the Mann-Whitney *U* test.
