# Supplementary material for: Neuronal metabotropic glutamate receptor 8 protects against neurodegeneration in CNS inflammation
Source: J Exp Med. 2021 Mar 4;218(5):e20201290. doi: 10.1084/jem.20201290 (PMC7938362; doi:10.1084/jem.20201290)
Supplement: Table S6 — summarizes clinical data for brain specimens. [file JEM_20201290_TableS6.docx]

Table S6. Clinical data for brain specimens

| Simplified name | Group | Details | Ag | Sex | Region | Note |
| --- | --- | --- | --- | --- | --- | --- |
| C1 | Control | NND | 55 | F | Frontal cortex |  |
| C2 | Control | NND | 43 | M | Frontal cortex |  |
| C3 | Control | NND | 45 | F | Frontal cortex |  |
| C4 | Control | NND | 68 | F | Frontal cortex |  |
| C5 | Control | NND | 66 | F | Occipital cortex |  |
| C6 | Control | NND | 64 | M | Occipital cortex |  |
| C7 | Control | NND | 45 | F | Cingular cortex |  |
| MS1 | MS | RRMS | 50 | F | Frontal cortex – inferior frontal gyrus |  |
| MS2 | MS | RRMS | 50 | F | Frontal cortex – superior frontal gyrus | Same patient as MS1 but different lesion |
| MS3 | MS | RRMS | 53 | F | Cingulate cortex |  |
| MS4 | MS | RRMS | 57 | F | Parietal cortex |  |
| MS5 | MS | RRMS | 50 | F | Occipital cortex | Same patient as MS1 but different lesion |
| NAGM1 | NAGM | NAGM | 54 | M | Occipital cortex |  |
| NAGM2 | NAGM | NAGM | 54 | M | Occipital cortex | Same patient as NAGM1 |
| NAGM3 | NAGM | NAGM | 45 | M | Posterior parietal cortex |  |
| NAGM4 | NAGM | NAGM | 50 | F | Frontal cortex – inferior frontal gyrus | Same patient as MS1 but NAGM – different block |
| NAGM5 | NAGM | NAGM | 50 | F | Frontal cortex – superior frontal gyrus | Same patient as MS1 but NAGM – different block |
| NAGM6 | NAGM | NAGM | 53 | F | Cingulate cortex | Same patient as MS3 |
| NAGM7 | NAGM | NAGM | 57 | F | Parietal cortex | Same patient as MS4 |
| EP1 | Epilepsy | Pharmacoresistent epilepsy | 9 | F | Temporal cortex |  |
| EP2 | Epilepsy | Pharmacoresistent epilepsy | 24 | M | Orbitofrontal lateral cortex |  |
| EP3 | Epilepsy | Pharmacoresistent epilepsy | 34 | F | Temporal cortex |  |
| EP4 | Epilepsy | Pharmacoresistent epilepsy | 35 | F | Parietal cortex |  |
| EP5 | Epilepsy | Epilepsy | 20 | F | Temporal cortex |  |

NND, no neurological disease; RRMS, relapsing-remitting MS.
