# Supplementary material for: Neuronal metabotropic glutamate receptor 8 protects against neurodegeneration in CNS inflammation
Source: J Exp Med. 2021 Mar 4;218(5):e20201290. doi: 10.1084/jem.20201290 (PMC7938362; doi:10.1084/jem.20201290)
Supplement: Table S7 — lists chemicals used in this study. [file JEM_20201290_TableS7.docx]

Table S7. Chemicals

| Compound | Chemical name | Company | Catalog no. | Concentration used in vitro | Function |
| --- | --- | --- | --- | --- | --- |
| 2-APB | 2-Aminoethoxydiphenylborane | Tocris | 1224 | 50 µM | Inhibitor of IP3Rs |
| AP5 | D-(-)-2-amino-5-phosphonopentanoic acid | Abcam | ab120003 | 100 µM | Inhibitor of NMDA glutamate receptor |
| AZ12216052 |  | Tocris | 4832 | 1 µM | Positive allosteric modulator of GRM8 |
| (+)-Bicuculline |  | Sigma-Aldrich | 14340 | 25 µM | Inhibitor of GABA-A receptor |
| Caffeine |  | Tocris | 2793 | 500 µM | Unspecific calcium release from ER |
| CGP 55845 | (2S)-3-[[(1S)-1-(3,4-Dichlorophenyl)ethyl]amino-2-hydroxypropyl](phenylmethyl)phosphinic acid hydrochloride | Tocris | 1248 | 2 µM | Inhibitor of GABA-B receptor |
| CNQX | 6-Cyano-7-nitroquinoxaline-2,3-dione | Sigma-Aldrich | C127 | 20 µM | Inhibitor of AMPA and kainate glutamate receptors |
| DAPI | 4′,6-Diamidino-2-phenylindole | Invitrogen | D1306 | 5 µM | Cell-impermeable DNA-binding dye |
| Forskolin |  | Tocris | 1099 | 10 µM | Activator of adenylyl cyclase |
| Glutamate | L-Glutamic acid monosodium salt monohydrate | Sigma-Aldrich | 49621 | variable: 1–100 µM | Excitatory amino acid and neurotransmitter |
| IBMX | 3-Isobutyl-1-methylxanthine | Sigma-Aldrich | I7018 | 50 µM | Inhibitor of phosphodiesterase |
| IFN-γ | Interferon-gamma | PeproTech | 315-05 | 100 ng/ml | Cytokine |
| Ionomycin | Ionomycin calcium salt | Alomone Labs | I-700 | 8 µM | Ionophore |
| NBQX | 2,3-Dioxo-6-nitro-1,2,3,4-tetrahydrobenzo[f]quinoxaline-7-sulfonamide disodium salt | Abcam | ab120046 | 20 µM | Inhibitor of AMPA and kainate glutamate receptors |
| PKI (5-24) | Protein kinase A inhibitor | Tocris | 6221 | 50 nM | Inhibitor of protein kinase A |
| TBOA | DL-threo-β-benzyloxy aspartic acid | Tocris | 1223 | 50 µM | Inhibitor of excitatory amino acid transporters |
| Thapsigargin |  | Enzo Life Sciences | BML-PE180-0001 | 1 µM | Inhibitor of SERCA, inhibits ER refilling |
| TNF-α | Tumor necrosis factor-alpha | PeproTech | 315-01A | 50 ng/ml | Cytokine |
| U73122 |  | Tocris | 1268 | 1.25 µM | Inhibitor of PLC |

GABA, γ-aminobutyric acid.
