# Supplementary material for: Neuronal metabotropic glutamate receptor 8 protects against neurodegeneration in CNS inflammation
Source: J Exp Med. 2021 Mar 4;218(5):e20201290. doi: 10.1084/jem.20201290 (PMC7938362; doi:10.1084/jem.20201290)
Supplement: Table S8 — lists antibodies used in this study. [file JEM_20201290_TableS8.docx]

Table S8. Antibodies

| Antigen | Host species | Company | Catalog no. | Clone | Ig fraction | Fluorophore | Dilution ICC | Dilution IHC | Dilution IHP | Dilution FACS |
| --- | --- | --- | --- | --- | --- | --- | --- | --- | --- | --- |
| APP A4 | Mouse | MilliporeSigma | MAB348 |  | IgG |  |  |  | 1:3,000 |  |
| B220 | Rat | BD Biosciences | 561226 | RA3-6B2 | IgG | V500 |  |  |  | 1:100 |
| Bassoon | Mouse | Enzo Life Sciences | SAP7F407 |  | IgG |  | 1:1,000 |  |  |  |
| BrdU | Mouse | BioLegend | 364108 | 3D4 | IgG | AF647 |  |  |  | 1:30 |
| CD3 | Rabbit | Abcam | Ab16669 |  | IgG |  |  |  | 1:100 |  |
| CD3ε | Armenian hamster | BioLegend | 100305 | 145-2C11 | IgG | FITC |  |  |  | 1:100 |
| CD3ε | Armenian hamster | BioLegend | 100328 | 145-2C11 | IgG | PerCP-Cy5.5 |  |  |  | 1:100 |
| CD4 | Rat | BioLegend | 100447 | GK1.5 | IgG | BV711 |  |  |  | 1:400 |
| CD4 | Rat | BioLegend | 100453 | GK1.5 | IgG | BV785 |  |  |  | 1:100 |
| CD4 | Rat | BioLegend | 100406 | GK1.5 | IgG | FITC |  |  |  | 1:1,000 |
| CD8a | Rat | BioLegend | 100750 | 53-6.7 | IgG | BV785 |  |  |  | 1:200 |
| CD8a | Rat | BioLegend | 100728 | 53-6.7 | IgG | PB |  |  |  | 1:100 |
| CD8a | Rat | BioLegend | 100728 | 53-6.7 | IgG | PE-Cy7 |  |  |  | 1:200 |
| CD11b | Rat | BioLegend | 101205 | M1/70 | IgG | FITC |  |  |  | 1:100 |
| CD11b | Rat | BioLegend | 101228 | M1/70 | IgG | PerCP-Cy5.5 |  |  |  | 1:400 |
| CD11c | Armenian hamster | BioLegend | 117309 | N418 | IgG | APC |  |  |  | 1:100 |
| CD11c | Armenian hamster | BioLegend | 117318 | N418 | IgG | PE-Cy7 |  |  |  | 1:300 |
| CD40 | Rat | BioLegend | 124612 | 3/23 | IgG | APC |  |  |  | 1:100 |
| CD45 | Rat | BioLegend | 103127 | 30-F11 | IgG | AF700 |  |  |  | 1:100 |
| CD69 | Armenian hamster | BioLegend | 104530 | H1.2F3 | IgG | BV605 |  |  |  | 1:50 |
| CD80 | Armenian hamster | BioLegend | 104708 | 16-10A1 | IgG | PE |  |  |  | 1:100 |
| CD86 | Rat | BioLegend | 105028 | GL-1 | IgG | PerCP-Cy5.5 |  |  |  | 1:100 |
| CD107b | Rat | Abcam | 553322 |  | IgG |  |  |  | 1:100 |  |
| GFP | Chicken | Abcam | Ab13970 |  | IgY |  | 1:1,000 |  |  |  |
| Grm8 | Rabbit | Abcam | Ab53094 |  | IgG |  | 1:100 | 1:100 |  |  |
| Grm8 | Mouse | Santa Cruz Biotechnology | Sc-517124 |  | IgG |  | 1:100 | 1:100 |  |  |
| I-A/I-E | Rat | BioLegend | 107620 | M5/114.15.2 | IgG | PB |  |  |  | 1:400 |
| Ig chicken | Donkey | Jackson ImmunoResearch | 703-545-155 |  |  | AF488 | 1:600 | 1:500 |  |  |
| Ig chicken | Donkey | Jackson ImmunoResearch | 703-606-155 |  |  | AF647 | 1:600 | 1:500 |  |  |
| Ig chicken | Goat | Abcam | Ab97145 |  |  | Cy3 | 1:600 | 1:500 |  |  |
| Ig guinea pig | Goat | Abcam | Ab150187 |  |  | AF647 | 1:600 |  |  |  |
| Ig mouse | Goat | PerkinElmer | NEF822001EA |  |  |  |  |  |  |  |
| Ig mouse | Donkey | Abcam | Ab150111 |  |  | AF647 | 1:600 | 1:500 |  |  |
| Ig mouse | Donkey | Abcam | Ab175658 |  |  | AF647 | 1:600 |  |  |  |
| Ig rabbit | Donkey | Abcam | Ab150105 |  |  | AF647 | 1:600 | 1:500 |  |  |
| Ly6G | Rat | BD Biosciences | 560601 | 1A8 | IgG | PE-Cy7 |  |  |  | 1:100 |
| Map2 | Chicken | Abcam | Ab5392 |  | IgY |  | 1:1,000 |  |  |  |
| NeuN | Mouse | Millipore | MAB377 | A60 | IgG |  |  | 1:100 |  |  |
| NeuN | Chicken | Millipore | ABN91 |  | IgG |  | 1:1,000 | 1:300 |  | 1:500 |
| NeuN | Rabbit | Abcam | Ab190565 |  | IgG | AF647 |  |  |  | 1:500 |
| NK1.1 | Mouse | eBioscience | 12-5941-82 | PK136 | IgG | PE |  |  |  | 1:100 |
| pCREB | Rabbit | Millipore | 06-519 |  | IgG |  | 1:500 | 1:500 |  | 1:2,000 |
| Psd-95 | Mouse | Millipore | MAB1596 |  | IgG |  | 1:200 |  |  |  |
| dsRed | Rabbit | Clontech | 632496 |  | IgG |  | 1:500 |  |  |  |
| SMI-31 | Mouse | BioLegend | SMI-31R |  | IgG |  |  | 1:1,000 |  |  |
| SMI-32 | Mouse | BioLegend | SMI-32P |  | IgG |  |  | 1:1,000 |  |  |
| Synapsin-1/2 | Guinea pig | Synaptic Systems | 106004 |  | IgG |  | 1:200 |  |  |  |
