# Supplementary material for: Neuronal metabotropic glutamate receptor 8 protects against neurodegeneration in CNS inflammation
Source: J Exp Med. 2021 Mar 4;218(5):e20201290. doi: 10.1084/jem.20201290 (PMC7938362; doi:10.1084/jem.20201290)
Supplement: Table S9 — lists primers and oligonucleotides that we used for creating overexpression constructs. [file JEM_20201290_TableS9.docx]

Table S9. Primers and oligonucleotides

| Name | Entity | Restriction site | Sequence |
| --- | --- | --- | --- |
| Primer_f_1 | Primer | BsiWI | 5′-TAGCGTACGGTGAGCAAGGGCGAGGAGCT-3′ |
| Primer_r_2 | Primer | Age | 5′-TAGACCGGTCTTGTACAGCTCGTCCATGCCG-3′ |
| Primer_f_3 | Primer | Age | 5′-TAGACCGGTCAGGAGTATGCGCATTCCATCC-3′ |
| Primer_r_4 | Primer | SalI | 5′-TAGGTCGACTCAGATTGAATGATTACTGTAGCTGATG-3′ |
| Primer_f_5 | Primer | SacI | 5′-TAGGAGCTCATGGTTTGTGAGGGAAAGCGCTC-3′ |
| Oligo_f_1 | Oligo | KpnI; BsiWI | 5′-CGCCACCATGGTTTGTGAGGGAAAGCGCTCAACCTCTTGCCCTTGTTTCTTCCTTTTGACTGCCAAGTTCTACTGGATCCTCACAATGATGCAAAGAACTCACAGCC-3′ |
| Oligo_r_2 | Oligo | KpnI; BsiWI | 5′-GTACGGCTGTGAGTTCTTTGCATCATTGTGAGGATCCAGTAGAACTTGGCAGTCAAAAGGAAGAAACAAGGGCAAGAGGTTGAGCGCTTTCCCTCACAAACCATGGTGGCGGTAC-3′ |
| Oligo_f_3 | Oligo | KpnI; EcoRI | 5′-CGATTCCTAGGTCGCTAGCCTAGACGTACGTTACATTGCGCGGCCGCTGAGCGAGAGCTCATGAGATCGTAGTCGACAGTCAGTCGCTTAAGTGATACAGTCGAG-3′ |
| Oligo_r_4 | Oligo | KpnI; EcoRI | 5′-AATTCTCGACTGTATCACTTAAGCGACTGACTGTCGACTACGATCTCATGAGCTCTCGCTCAGCGGCCGCGCAATGTAACGTACGTCTAGGCTAGCGACCTAGGAATCGGTAC-3′ |
